# Two novel phages infecting *Erythrobacter* isolated from the epipelagic ocean

**DOI:** 10.3389/fmicb.2025.1592355

**Published:** 2025-06-10

**Authors:** Longfei Lu, Xingyu Huang, Pengfei Zheng, Shuzhen Wei, Nianzhi Jiao, Rui Zhang, Xuejing Li, Yunlan Yang

**Affiliations:** ^1^Key Laboratory of Tropical Marine Ecosystem and Bioresource, Fourth Institute of Oceanography, Ministry of Natural Resources, Beihai, China; ^2^Carbon Neutral Innovation Research Center, State Key Laboratory of Marine Environmental Science, Fujian Key Laboratory of Marine Carbon Sequestration, College of Ocean and Earth Sciences, Xiamen University, Xiamen, China; ^3^Institute of Tibetan Plateau Research, Chinese Academy of Sciences, Beijing, China; ^4^Laboratory of Beibu Gulf Ocean Big Data Application, Fourth Institute of Oceanography, Ministry of Natural Resources, Beihai, China; ^5^State Key Laboratory of Marine Geology, Tongji University, Shanghai, China; ^6^Archaeal Biology Center, Synthetic Biology Research Center, Shenzhen Key Laboratory of Marine Microbiome Engineering, Key Laboratory of Marine Microbiome Engineering of Guangdong Higher Education Institutes, Institute for Advanced Study, Shenzhen University, Shenzhen, China

**Keywords:** bacteriophage, *Erythrobacter*, biological features, genome, new genus

## Abstract

*Erythrobacter*, an aerobic anoxygenic photoheterotrophic bacterial genus, plays a vital role in carbon and energy cycling in marine environments. However, their phage predators remain poorly understood, with only two strains previously reported. This study isolated and characterized a novel *Erythrobacter* phage, vB_EauS-R34L1 (R34L1), and its sub-strain vB_EauS-R34L2 (R34L2), from coastal seawater. Both phages exhibit long-tailed, icosahedral morphologies and relatively narrow but slightly different host ranges. One-step growth curve analysis revealed a 160-min latent period and burst sizes of 81 and 91 PFU/cell for R34L1 and R34L2, respectively. Genomic analysis showed that the phages possess dsDNA genomes of 56,415 bp (R34L1) and 54,924 bp (R34L2), with G + C contents of 61.60 and 61.19%, respectively. Both phages harbor a suite of unique genes, including GapR and GH19, which are crucial for host interaction and ecological functionality. Blastn analysis indicated a 99.73% genome similarity between them. Taxonomic and phylogenetic analyses positioned them in a novel viral genus cluster, *Eausmariqdvirus*, under the family *Casjensviridae*, indicating a distant evolutionary relationship with known phages. Metagenomic queries suggested that R34L1- and R34L2-like phages are exclusively abundant in temperate and tropical epipelagic zones. This study expands our understanding of *Erythrobacter* phages and provides insights into their ecological roles in marine ecosystems.

## 1 Introduction

Aerobic anoxygenic photoheterotrophic bacteria (AAPB), which contain bacteriochlorophyll-*a* and lack light-harvesting complex II, represent photoheterotrophic microorganisms that may consume less organic carbon and be crucial to the marine carbon cycle (Eiler, 2006; Jiao et al., 2007; Kolber et al., 2001; Zhang et al., 2016). *Erythrobacter* is the first identified AAPB and frequently detected in and isolated from nutrient-rich coastal seawaters and sediments, and its metabolism is versatile (Jeong et al., 2022; Lei et al., 2015; Shiba and Simidu, 1982; Yurkov et al., 1994; Zheng et al., 2016; Zhuang et al., 2015). Studies have demonstrated that *Erythrobacter* species show potential applications in bioremediation of alkane contamination (e.g., *E. longus* and *E. citreus*) (RoLing et al., 2002), production of yellow xanthophyll pigment (e.g., *Erythrobacter* sp. SDW2) (Jeong et al., 2022), and production of poly-*β*-hydroxybutyrate, which is known as a material for degradable plastics (e.g., *E. aquimaris*) (Mostafa et al., 2020). *Erythrobacter* has also shown capacities for nitrate reduction, denitrification, aesculin hydrolysis, and multiple substrates utilization, such as amino acids, carbohydrates, and fatty acids (Fang et al., 2019; Koblížek et al., 2003; Wei et al., 2013; Zhuang et al., 2015), and refractory dissolved organic carbon degradation, and may play an essential role in labile dissolved organic carbon acquisition for surrounding heterotrophs (Zhang et al., 2016; Zhuang et al., 2015). While *Erythrobacter*’s metabolic versatility and potential applications in bioremediation and biotechnology are well-documented, our understanding of the phages that infect them remains limited.

Bacteriophages (viruses that infect bacteria, phage for short) are crucial components of marine ecosystems. They could regulate bacterial abundance, community structure, and carbon sequestration efficiency (Jiao et al., 2014; Suttle, 2007; Weinbauer, 2004). The isolation and identification of the genomic features of phages could advance our knowledge of the ecology and evolution of their hosts. To date, nearly 80 roseophages—viruses that infect the *Roseobacter* lineage (a representative member of AAPB), have been identified and are suggested to provide versatility for roseobacters to adapt to changing environments quickly (Huang et al., 2025; Zhan and Chen, 2019). They can rapidly alter the growth and abundance of their hosts to shunt their secondary production (Huang et al., 2011). Similarly, cyanophages (viruses that infect cyanobacteria) can mediate the mortality of cyanobacteria and affect their distribution and composition (Proctor and Fuhrman, 1990; Suttle, 2005). Beyond that, the isolation of novel phages could also contribute to understanding the ecological distribution patterns and physiological characteristics of phages in aquatic environments by combining the virome database (Budinoff, 2012; Lu et al., 2017; Yang et al., 2021). Despite advancements in phage research targeting other marine bacteria like *Roseobacter* and cyanobacteria, knowledge of *Erythrobacter* phages is still fragmented.

To date, only two *Erythrobacter* phages, vB_EliS-R6L (hereafter R6L) and vB_EliS-L02 (hereafter L02) have been isolated, each exhibiting unique morphological traits and distinct ecological distribution patterns (Li et al., 2022; Lu et al., 2017). These phages exhibit complex interactions with their hosts, highlighting their ecological and evolutionary significance. For example, both phages contain *phoH* genes, which promote host phosphorus uptake in low-phosphorus environments. Additionally, phage L02 harbors several genes related to nucleotide metabolism, potentially enhancing its ability to regulate host metabolic processes. Despite these advances, our understanding of *Erythrobacter* phages remains fragmented, particularly concerning their morphology diversity, physiological features, ecological distribution patterns, and interactions with the host. Here, we reported one novel *Erythrobacter* phage and its sub-strain, providing detailed insights into their morphology, basic physiology, genomic features, and distribution patterns, which represent a significant step forward in the study of *Erythrobacter* phages and offer a foundation for future research into their ecological functions and host-phage coevolutionary relationships.

## 2 Materials and methods

### 2.1 Isolation and propagation of phages

*Erythrobacter* sp. JL 475, isolated from seawater samples in the South China Sea at a depth of 75 m (20.00 °N, 112.00 °E) (Zheng et al., 2016), was used as a host to detect phages and evaluate their physical features. All of the bacterial strains used in this study are listed in Supplementary Table S1. RO medium (containing 1 g/L yeast extract, 1 g/L tryptone, and 1 g/L sodium acetate, pH 7.5) was used for bacterial cultivation (Yurkov et al., 1999).

Phages vB_EauS-R34L1 and vB_EauS-R34L2 were separately isolated from seawater obtained at the coast of Zhoushan (30.05 °N, 122.35 °E) in September 2013 and Qingdao (36.06 °N, 120.32 °E) in February 2014, China, respectively. Twenty milliliters of surface seawater, filtered through a 0.22 μm filter membrane (Millipore, USA), was added to 100 mL of *Erythrobacter* sp. JL 475 culture (mid-log phase) and incubated overnight at 30°C. The mixture was then filtered to remove bacterial cells. The filtrate was subsequently diluted serially to isolate phages using the double-agar layer method (Pajunen et al., 2000). Each single plaque was purified at least three times using sodium chloride-magnesium sulfate (SM) buffer (100 mM NaCl, 50 mM Tris, 10 mM MgSO_4_, and 0.01% gelatin, pH 7.5) with several drops of chloroform and stored at −80°C.

For phage propagation, the phage suspension was added to the host cultures (mid-log phase) at a multiplicity of infection (MOI) of 10. The mixture was then incubated at 30°C for 12 h with shaking. Afterward, the sample was centrifuged at 6,000 × g for 10 min and filtered to remove bacterial cells. Then, the filtrate was treated with 10% (w/v) Polyethylene glycol (PEG) 8,000 (containing 1 M NaCl) and incubated at 4°C overnight. The phage particles were subsequently concentrated using CsCl density gradient ultracentrifugation (Zheng et al., 2018). Finally, the purified phage particles were dialyzed twice in SM buffer to remove CsCl.

### 2.2 Transmission electron microscopy (TEM)

Phage morphology was analyzed using transmission electron microscopy (TEM). The purified phage suspension was placed on a copper grid and negatively stained with 2% uranyl acetate for 10 min. Then the grid was examined under a 120 kV TEM (JEM-2100HC transmission electron microscope, JEOL, Japan). The isolated phages were identified and classified according to the guidelines of the International Committee on Taxonomy of Viruses (Murphy et al., 2012). The phage sizes were calculated from at least 10 particles.

### 2.3 Chloroform sensitivity

Filtered lysate (~10^9^ plaque-forming units (PFU)/mL) was mixed with chloroform at a final concentration of 0, 1, 10, and 50%, separately. Each mixture was then vigorously shaken for 2 min. After incubation at 30°C for 30 min, the samples were diluted and plated for phage titration using the double-layer agar method. All assays were carried out in triplicate.

### 2.4 Host range test

The lytic spectra of phages were determined using the double-layer agar method. The host range of isolated phages was evaluated against 50 bacterial strains, including 31 *Erythrobacter* strains, 17 *Citromicrobium* strains, one *Dinoroseobacter* strain, and one *Roseobacter* strain. The testing was performed by spotting serial dilutions of the phage suspensions (~10^7^, ~10^8^, ~10^9^ PFU/mL) onto double-layer agar plates that had been previously inoculated with the potential host strains. The plates were then incubated at 30°C for at least 24 h. After incubation, the presence of phage plaques on the bacterial lawns was examined to determine the host range of the isolated phages. All assays were carried out in triplicate.

### 2.5 One-step growth curve

One-step growth curves were performed to determine the burst size and latent period of the isolated phages, following previously described methods (Pajunen et al., 2000; Wu et al., 2007). Briefly, each phage was inoculated separately into 100 mL of the host culture (optical density at 600 nm = 0.3 ~ 0.5) at an MOI of 0.1. For phage adsorption, each mixture was incubated for 10 min. After incubation, the mixture was centrifuged at 8,000 × g for 5 min. The cell pellet was resuspended in RO medium and cultured at 30°C. The phage titer was measured every 20 min for a total of 220 min post-infection. Triplicate samples were serially diluted and titrated by the double-layer plaque assay method (Pajunen et al., 2000). The latent period and burst size were calculated based on the ratio between the phage count at the post-burst plateau and the initial phage count (Yang et al., 2017).

### 2.6 Stability characterization

To determine the effect of pH on phage stability, the isolated phages were diluted in SM buffer at different pH values (ranging from pH 5 to 9). The pH was adjusted and stabilized using 5 M HCl, 0.2 M Na₂HPO₄/NaH₂PO₄ buffer, or 5 M NaOH. The diluted phages were then incubated at 30°C for 24 h. The thermal tolerance of the isolated phages was tested by incubating the phages in SM buffer at various temperatures (4°C, 8°C, 12°C, 16°C, 20°C, 24°C, 30°C, 32°C, 36°C, and 40°C) for 24 h. After incubation, the phage suspensions were cooled to 4°C to estimate phage activity. The salinity stability of the isolated phages was assessed by incubating the phages in mixtures of sterile seawater (34‰) and sterile freshwater for 24 h, with varying percentages (0 to 100%) of seawater. Phage activity was determined using the double-layer plaque assay method (Pajunen et al., 2000). All the experiments were repeated at least three times.

### 2.7 Phage DNA extraction, genome sequencing and bioinformatics

Phage DNA was extracted using the phenol/chloroform DNA extraction method. Briefly, the CsCl-purified phage was first treated with DNase I (1 μg/mL) and Rnase A (1 μg/mL) for 30 min at 37°C to remove free DNA and RNA. Subsequently, the mixture was treated with 100 μg/mL Proteinase K and 10% SDS for 2 h at 56°C. Then, one volume of phenol/chloroform/isoamyl alcohol (25:24:1) was added and centrifuged for 10 min at 12,000 rpm. The aqueous layer was collected and extracted with an equal volume of chloroform/isoamyl alcohol (24:1). After centrifugation for 10 min at 12,000 rpm, DNA was precipitated by adding isopropanol (1:1) and sodium acetate (10:1) for 1 h at −20°C. The mixture was then centrifuged for 10 min at 12,000 rpm to pellet the DNA. Next, the DNA pellet was washed twice with 70% and once with 100% ethanol. Finally, the DNA was dried and resuspended in 50 μL 10 mM Tris (pH = 8.0), and concentration was measured using a Qubit fluorimeter (Life Technologies, USA).

The phage genomes were sequenced using the Illumina Miseq platform with 2 × 250 bp paired-end reads. The raw reads were assembled using CLC Genome Workbench software (43 × coverage). The quality and completeness of the assembled genomes were determined by CheckV with default parameters (Nayfach et al., 2021). The putative open reading frames (ORFs) were predicted with GeneMarkS online server (http://exon.gatech.edu/Genemark/genemarks.cgi) (Besemer et al., 2001), Glimmer 3.0 (http://ccb.jhu.edu/software/glimmer/index.shtml) (Delcher et al., 2007), and ORF Finder online server (https://www.ncbi.nlm.nih.gov/orffinder/). The predicted ORFs were then annotated against the GenomeNet nr-aa database (a non-redundant protein sequence database merging sequences from RefSeq, SwissProt, TrEMBL, and GenPept), with a cut-off E-value of 10^−5^ (Nishimura et al., 2017). The final annotation of the ORFs was manually verified to ensure accuracy. The tRNA was identified using tRNAscan-SE 2.0 (http://lowelab.ucsc.edu/tRNAscan-SE/) (Chan and Lowe, 2019; Lowe and Chan, 2016). The genomic structures of the two isolated phages were conducted using CGView-Circular Genome Viewer (https://proksee.ca) (Grant and Stothard, 2008). The sequence data of the isolated phages R34L1 and R34L2 have been deposited in the GenBank databases under access No. PQ394074 and PQ394075, separately.

### 2.8 Phylogenetic analysis

The Viral Proteomic Tree server (ViPTree, https://www.genome.jp/viptree/) was used to employed to generate a proteomic tree based on genome-wide sequence similarities, computed by tBLASTx (Nishimura et al., 2017; Rohwer and Edwards, 2002). Subsequently, the average nucleotide identity by orthology (OrthoANI) values were performed by the OAT software V0.93 (Lee et al., 2016). Then, the phage genome similarities were calculated using the VIRIDIC tool, which employs BLASTn with default parameters (https://rhea.icbm.uni-oldenburg.de/VIRIDIC/) (Moraru et al., 2020). The taxonomy position of the isolated phages was further investigated with Virus Classification and Tree Building Online Resource (VICTOR; https://ggdc.dsmz.de/victor.php) (Meier-Kolthoff and Göker, 2017), employing the Genome-BLAST Distance Phylogeny (GBDP) method under settings recommended for prokaryotic viruses (Meier-Kolthoff et al., 2013). BLASTx was employed to assess the similarity of ORFs among phages R34L1 and R34L2 and their respective closest phages, with a cut-off E-value of 10^−5^ (Altschul et al., 1997).

The neighbor-joining phylogenetic trees were constructed using glycoside hydrolase family 19 (GH19), major capsid protein, and portal protein. The seed amino acid sequences of GH19 used for phylogenetic analysis were retrieved from NCBI non-redundant protein database (https://www.ncbi.nlm.nih.gov/refseq/about/nonredundantproteins/) and the Protein Data Bank (PDB, https://www.rcsb.org/) with a maximal E-value of 10^−10^ based on Orlando et al. (2021). Amino acid sequences of orthologs of the major capsid protein and portal protein were retrieved from NCBI by BLASTp with the nr protein database. Sequence alignments and phylogenetic analyses were performed using MEGA X software with 1,000 bootstrap replications to assess the robustness of the tree topology (Kumar et al., 2018).

### 2.9 Recruitment of reads to metagenomic data

To evaluate the distribution of phages R34L1 and R34L2 in different marine environments, the genomes of the isolated phages and their related phages, previous *Erythrobacter* phages, *Citromicrobium* phages were mapped to the Global Ocean Viromes 2.0 (GOV 2.0) (Gregory et al., 2019) using minimap2 (2.17-r941) (Li, 2018). The relative abundance of the phages was compared using Transformed Per Million (TPM) mapped reads (Vera Alvarez et al., 2019). To ensure the accuracy of the analysis, several widely distributed representative phages were included as references, namely *Pelagibacter* phages HTVC010P, HTVC019P, and HTVC011P, and Cyanophages P-SSP7, P-SSM7, P-HM1, P-HM2, S-SSM7, S-B28, and S-B05. The relative abundance of these phages was calculated using CoverM (v0.3.1) (Zhang et al., 2023) and visualized by R packages through the OmicStudio platform (Lyu et al., 2023).

## 3 Results

### 3.1 Phage isolation and biological features

**Figure 1:**
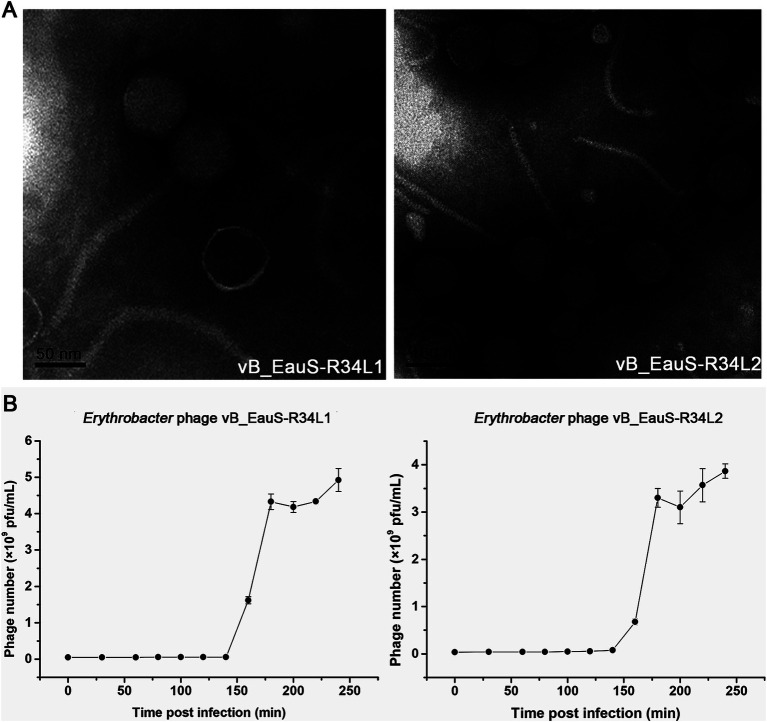
Isolation and growth curve of phages vB_EauS-R34L1 and vB_EauS-R34L2. **(A)** Transmission electron microscopy (TEM) results. **(B)** One-step growth curve. Error bars indicate standard deviations among triplicate samples.

Two phages, vB_EauS-R34L1 (hereafter R34L1) and vB_EauS-R34L2 (hereafter R34L2), infecting *Erythrobacter* sp. JL 475, were isolated from the Qingdao and Zhoushan coastal seawater, respectively. Both phages formed small, clear, and round plaques on the lawn of *Erythrobacter* sp. JL 475 with a diameter of approximately 3 ~ 4 mm. Transmission electron microscopy (TEM) analysis revealed that phages R34L1 and R34L2 both belonged to siphovirus (Figure 1A), exhibiting icosahedral capsids with average diameters of 56.7 ± 3.0 and 57.0 ± 2.1 nm, and visible long tails with average lengths of 224.6 ± 14.0 nm and 188.5 ± 3.6 nm, respectively. Additionally, chloroform treatment analysis revealed that phages R34L1 and R34L2 were not sensitive to chloroform, suggesting that these phages do not contain lipids as structural components, as lipids are typically dissolved by chloroform (Espejo and Canelo, 1968).

Both phages showed a relatively narrow host range (Supplementary Table S1). Specifically, phage R34L1 could only infect *Erythrobacter* sp. JL 475, while phage R34L2 lysed *Erythrobacter* sp. JL 475, JL 967 and JL 2286. Notably, the host ranges of isolated phages differ from those of phages R6L and L02 (Li et al., 2022; Lu et al., 2017).

The infection activities of phages R34L1 and R34L2 were characterized by a one-step growth curve derived from plaque assays. Both phages exhibited latent and rising periods of approximately 160 min (Figure 1B). The burst sizes reached approximately 91 and 88 plaque-forming units per infected cell (PFU/cell) for phages R34L1 and R34L2, respectively.

**Figure 2:**
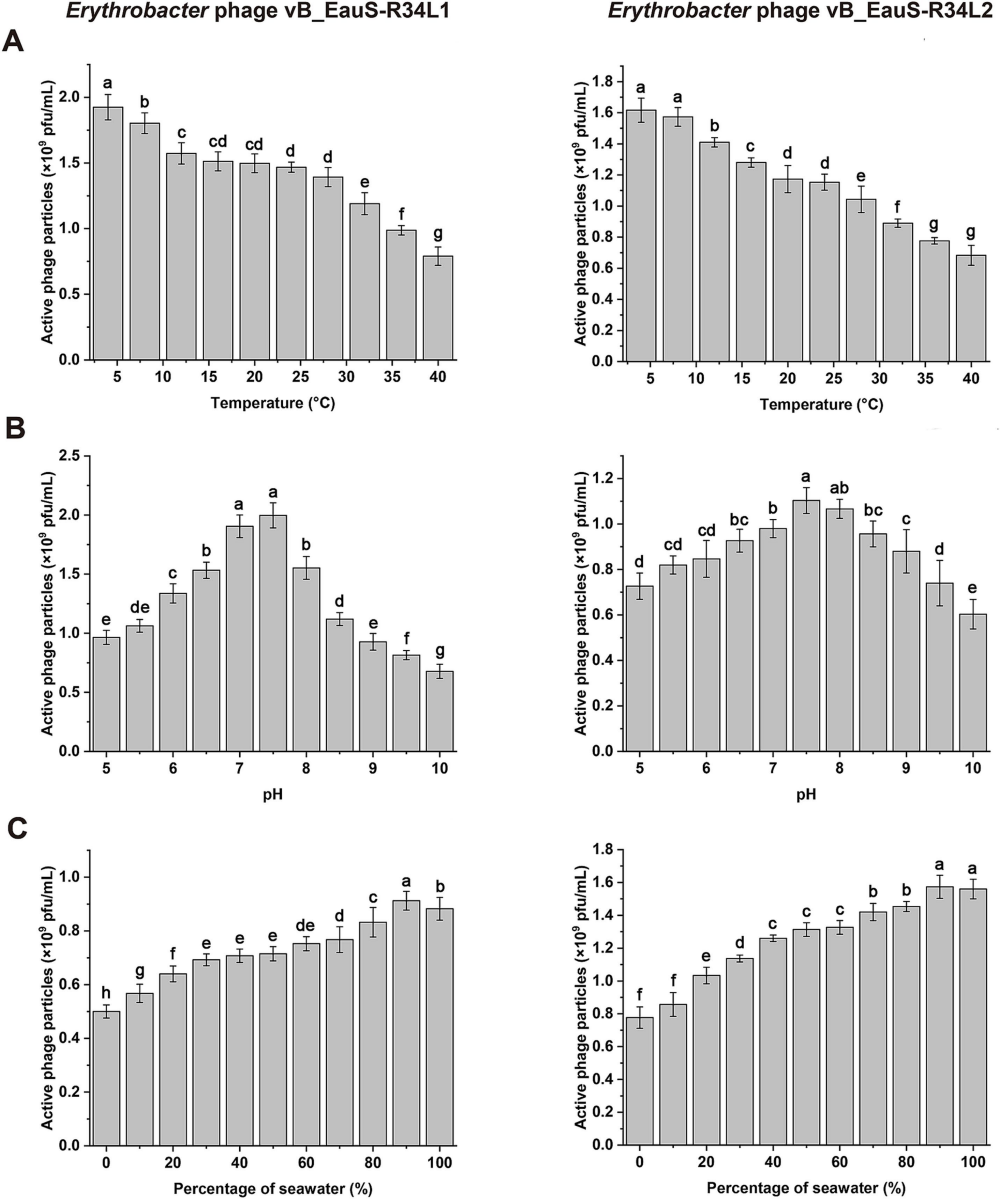
Environmental stability of phages vB_EauS-R34L1 and vB_EauS-R34L2. **(A)** Thermal stability treated with different temperatures for 24 h. **(B)** pH stability treated with different pH for 24 h. **(C)** Salinity stability treated with different salinity conditions for 24 h. Error bars indicate standard deviations among triplicate samples. Significant differences (ANOVA test, *p* < 0.05) among different treatments are represented by different letters.

The stability of phages R34L1 and R34L2 was evaluated across different temperatures, pH levels, and salinities. Overall, the trends in stability were similar for both phages. The activity of phages R34L1 and R34L2 was significantly affected by temperature, with a notable decrease in activity observed as the temperature increased (Figure 2A). pH stability tests revealed that the highest activity for both phages was at pH 7.5, indicating that these phages are sensitive to both acidic and alkaline conditions (Figure 2B). The salinity stability experiment demonstrated a decrease in phage viability with increasing freshwater dilution ratios, with viabilities of 52.49 and 49.58% in total freshwater for phages R34L1 and R34L2, respectively (Figure 2C).

### 3.2 Genomic features of R34L1 and R34L2

Phages R34L1 and R34L2 both possess dsDNA genomes, with sizes of 56,415 bp and 54,924 bp, respectively. Their G + C contents are 61.60 and 61.19%, respectively. Notably, the two genomes exhibit a high degree of similarity, with an identity of 99.73% as determined by Blastn and 99.68% by OrthoANI analysis. The G + C contents of both phage genomes are slightly lower than the G + C content of their host (61.77%, GenBank accession no. NZ_CP017057) and differ from those of other isolated *Erythrobacter* phages (59.43 and 66.52%) (Table 1) (Li et al., 2022; Lu et al., 2017). Bioinformatic analysis revealed that neither phage R34L1 nor R34L2 contains tRNA genes, which may also account for their relatively narrow host range (Chan and Lowe, 2019; Lowe and Chan, 2016).

**Table 1:** Genomic features of isolated *Erythrobacter* phages.

Phage name	Genome size (bp)	G + C%	ORFs	tRNA	Accession no.	Reference
vB_EauS-R34L1	56,415	61.60	78	0	PQ394074	This study
vB_EauS-R34L2	54,924	61.19	72	0	PQ394075	This study
vB_EliS-R6L	65,675	66.52	108	0	KY006853	Lu et al. (2017)
vB_ElisS-L02	150,063	59.43	61	29	OL955261	Li et al. (2022)

A total of 78 open reading frames (ORFs) were predicted for phage R34L1 and 72 ORFs for phage R34L2 (Supplementary Figure S1; Supplementary Tables S2, S3). Functional analysis revealed that 27 ORFs (34.62%) in phage R34L1 and 27 ORFs (37.50%) in phage R34L2 had predicted functions (Supplementary Tables S2, S3). Furthermore, the predicted ORFs between the two phages were largely consistent, with high similarity ranging from 94.57 to 100.00% as determined by Blastp analysis (Supplementary Table S4).

The identity values determined by Blastn, combined with subsequent Average Nucleotide Identity (ANI) and VIRIDIC analysis, are all higher than 95%, indicating that the two phages belong to the same species (Turner et al., 2021). Therefore, we propose that phage R34L2 is a sub-strain of R34L1. Subsequently, the introduction of functional genes primarily focuses on phage R34L1. The functionally related ORFs can be divided into four main groups: DNA replication and metabolism (8 ORFs), assembly and structure (17 ORFs), host lysis (ORF33, glycoside hydrolase family 19 catalytic domain-containing protein), and auxiliary metabolic genes (AMGs) (ORF28, GapR) (Supplementary Figure S1). Specifically, eight ORFs were related to DNA replication and metabolism, including DNA synthesis (ORF65, ORF66, and ORF72), DNA methyltransferase (ORF64), DNA helicase-related protein (ORF62), and ligase-related proteins (ORF39, ORF63, and ORF67). Seventeen ORFs were related to assembly and structure, including tail-related proteins (ORF37, ORF38, ORF39, ORF42, ORF43, ORF44, ORF47, ORF48, and ORF49), stopper protein (ORF50), major capsid protein (ORF52), head decoration protein (ORF53), S49 family peptidase (ORF54), portal protein (ORF55), head-tail adaptor protein (ORF54), and phage packaging protein terminase (ORF57 and ORF58).

### 3.3 Phylogenetic analysis and comparative genomic analyses

**Figure 3:**
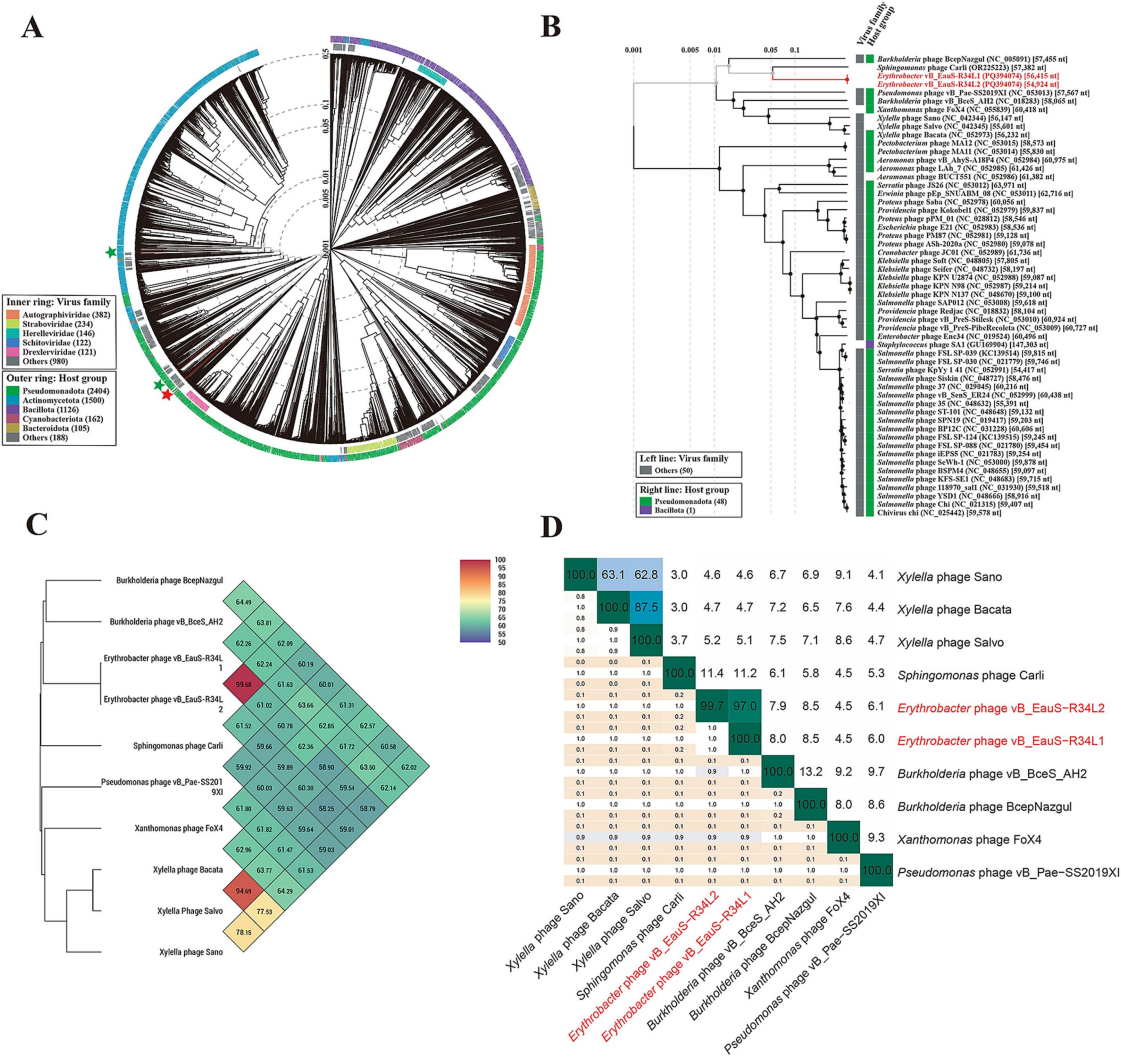
Phylogenetic and genomic comparison analysis. **(A)** Determination of taxa and host group of phages vB_EauS-R34L1 and vB_EauS-R34L2 by a proteomic tree using ViPTree server. The colored rings represent the virus family (inner ring) and host group (outer ring). The red star marks the position of phages vB_EauS-R34L1 and vB_EauS-R34L2, and the green star marks the position of phages vB_ElisS-R6L and vB_EliS-L02. **(B)** Phylogenetic relationship of phages vB_EauS-R34L1 and vB_EauS-R34L2 with their closest relatives. The left and right color bars indicate the taxonomic virus family and host group, respectively. **(C)** Heatmap showing OrthoANI values of phages vB_EauS-R34L1 and vB_EauS-R34L2 with their closest relatives. **(D)** The intergenomic comparison of phages vB_EauS-R34L1 and vB_EauS-R34L2 with their closest relatives. The heatmap generated by VICDIC shows the intergenomic similarity values (right half) and alignment indicators (left half).

The genomes of phages R34L1 and R34L2 were analyzed using Blastn on Genebank, and neither of them showed significant similarity to any of the uploaded sequences in the NCBI nr database, with query coverage ranging from 0 to 1% for all other alignment sequences (accessed on 15 February 2025). To assess the phylogenetic relationship of phages R34L1 and R34L2 to known phages, the ViPTree server was utilized to construct a proteomic tree based on genome sequences (Figures 3A,B). The results indicated that phages R34L1 and R34L2 only clustered with *Sphingomonas* phage Carli (OR225223.1) and *Burkholderia* phage BcepNazgul (NC_005091), yet exhibiting a relatively distant evolutionary relationship. Moreover, phages R34L1 and R34L2 and the taxa clustered with them were classified within the virus family “Others” (Figure 3B).

Subsequently, the phages clustered with phages R34L1 and R34L2 were selected for further genome comparison analysis. The ANI values between R34L1 or R34L2 were 99.68%, while they ranged from 58.91 to 61.37% when compared with other phages, suggesting that phages R34L1 and R34L2 may represent a new genus, based on mechanistic demarcation criterion for phage genera (70%) (Figure 3C) (Turner et al., 2021). Additionally, the genomic similarity between phages R34L1, R34L2 and other phages just ranged from 4.46 to 8.54%, as determined by VIRIDIC (Figure 3D).

**Figure 4:**
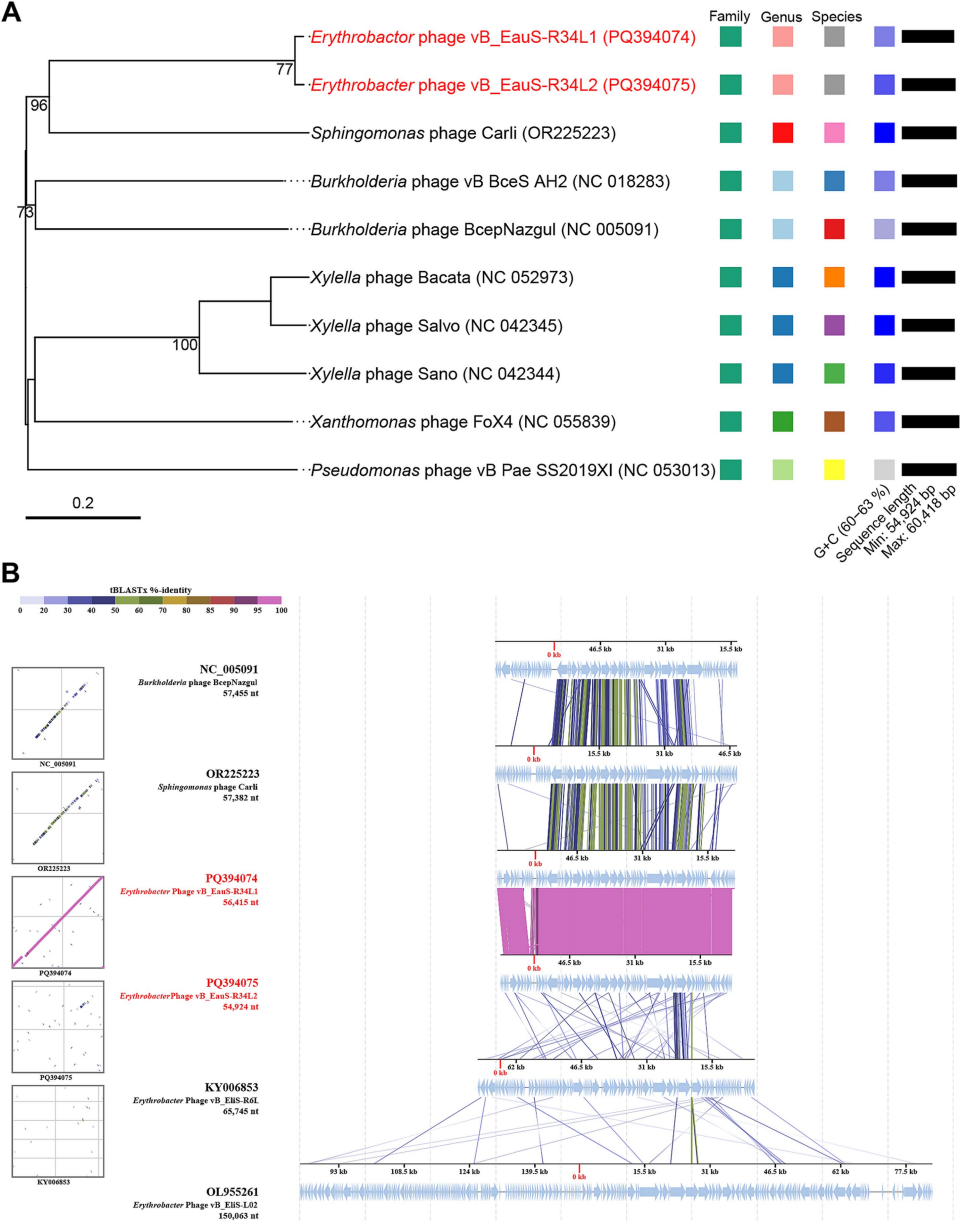
Phylogenetic analysis based on whole genome and conserved proteins. **(A)** Phylogenetic analysis of whole-genome sequences of phages vB_EauS-R34L1 and vB_EauS-R34L2 with their closest relatives. Annotations, including species, genus, and family cluster were predicted by VICTOR, with different colors, which are different in classification. **(B)** Genomic comparison between *Erythrobacter* phages vB_EauSS-R34L1, vB_EauSS-R34L2, vB_EliS-R6L and vB_EliS-L02, *Sphingomonas* phage Carli, and *Burkholderia* phage BcepNazgul. Arrows present the ORFs. The direction of each arrow represents the direction of transcription. Genome regions showing similarity were searched using tBLASTX and matches satisfying length and E-value (<10^−5^) cutoffs were indicated by the rectangle according to the color scale on the top.

Then, the phylogenetic tree constructed by VICTOR (https://ggdc.dsmz.de/victor.php#) also suggested that phages R34L1 and R34L2 exhibit strong novelty and should be classified as a new viral genus within the family *Casjensviridae*, with no other known members (Figure 4A). And, the comparative genomic analysis was performed with phages R34L1, R34L2, R6L, and L02, as well as *Sphingomonas* phage Carli and *Burkholderia* phage BcepNazgul (Figure 4B). Based on BLASTx analysis results, phage R34L1 or R34L2 shares 33 and 30 similar ORFs (*E*-value <10^−5^) with *Sphingomonas* phage Carli, and *Burkholderia* phage BcepNazgul, respectively, and six and four with R6L and L02, respectively. Only one homologous ORF, the tail fiber protein, is shared among all four *Erythrobacter* phages with an average identity of 50.29%. However, the identities of all pairs only ranged from 23.86 to 60.76% (with an average of 39.03%).

**Figure 5:**
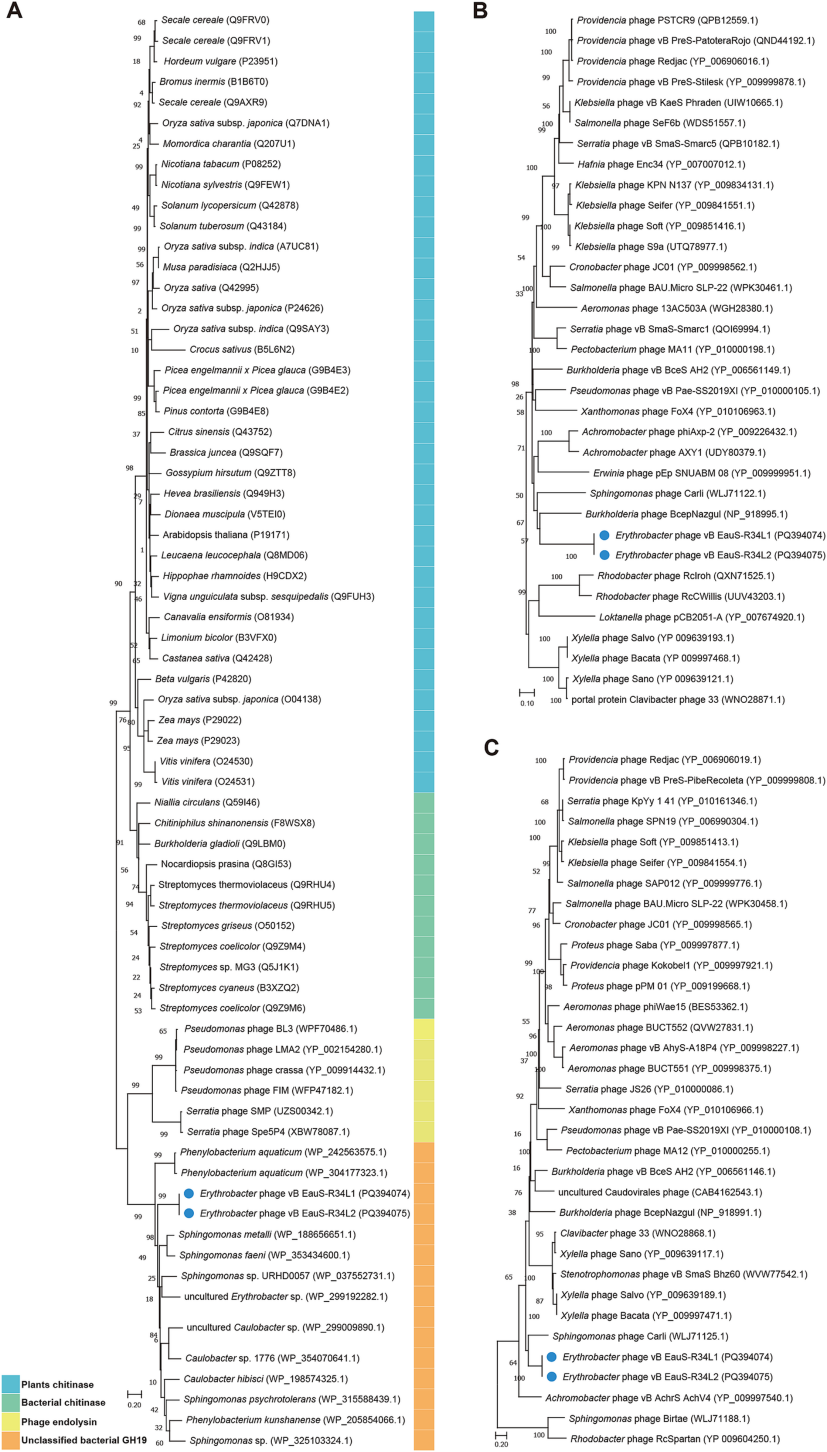
The neighbor-joining phylogenetic tree based on amino acid sequences. **(A)** Phylogenetic analysis using glycoside hydrolase family 19 catalytic domain-containing protein of phages vB_EauS-R34L1 and vB_EauS-R34L2 with seed sequences retrieved from NCBI non-redundant protein database and Protein Data Bank. **(B)** Phylogenetic analysis using the portal protein of phages vB_EauS-R34L1 and vB_EauS-R34L2 with their closest relatives. **(C)** Phylogenetic analysis using major capsid protein of phages vB_EauS-R34L1 and vB_EauS-R34L2 with their closest relatives. Bootstrap values were based on 1,000 replicates.

Next, the phylogenetic relationship between phages R34L1 and R34L2 and their related phages was further analyzed using GH19 protein (ORF 33), major capsid protein (ORF 52), and portal protein (ORF 55). Firstly, the distant phylogenetic relationships with other phages indicated that phages R34L1 and R34L2 have a significant genetic distance from other known phages (Figure 5). In the phylogenetic trees based on the portal protein and major capsid protein, phages R34L1 and R34L2 primarily clustered with *Sphingomonas* phage Carli, *Burkholderia* phage BcepNazgul, consistent with the result of the ViPTree analysis. However, in the GH19-based tree, phages R34L1 and R34L2 clustered with bacteria sequences, including *Erythrobacter*, *Sphingomonas*, *Caulobacter*, and *Phenylobacterium*, rather than with other phages.

### 3.4 Marine ecological distribution of R34L1 and R34L2

**Figure 6:**
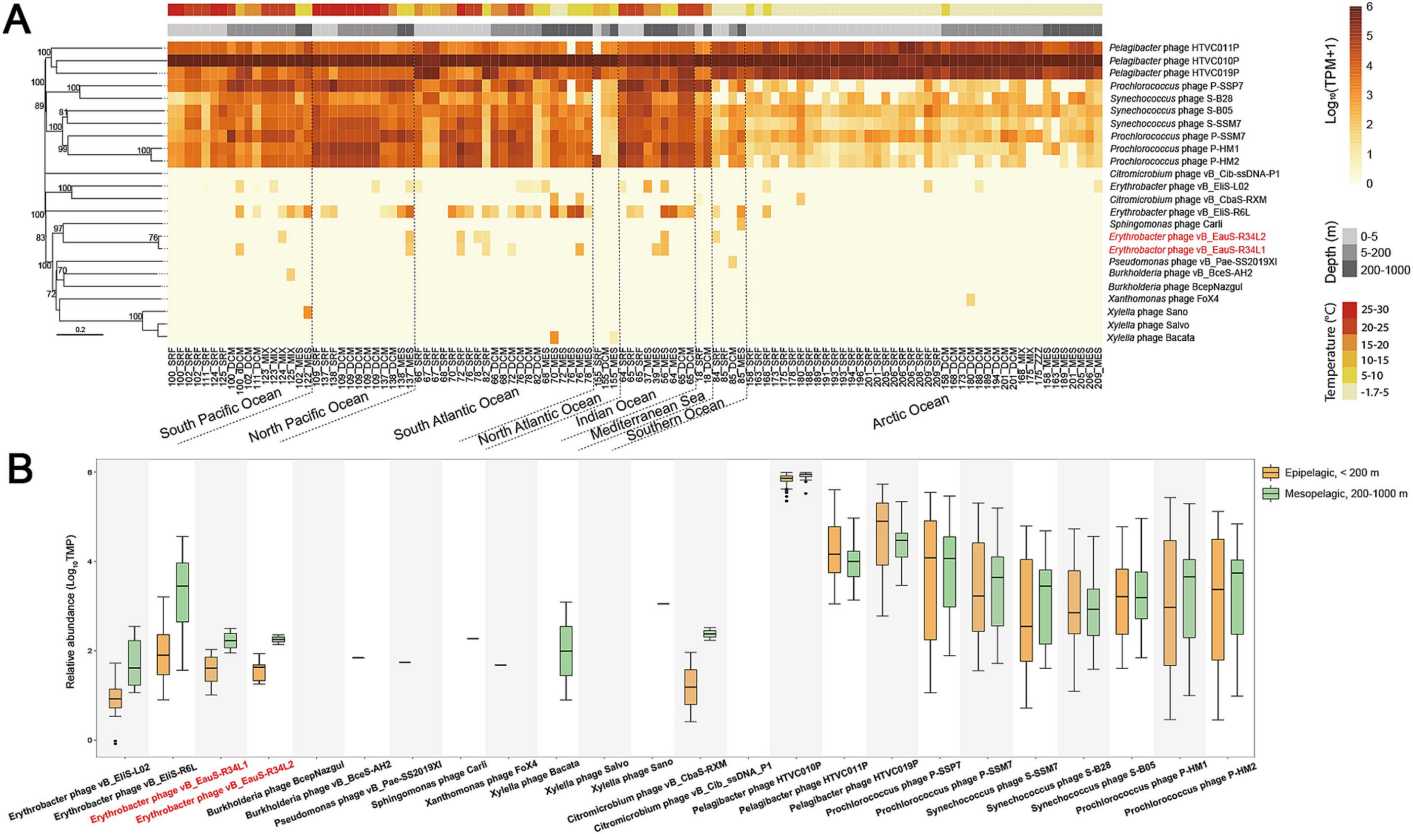
Relative abundance of phages vB_EauS-R34L1 and vB_EauS-R34L2 compared to the abundance of phylogenetically related phages, two published *Erythrobacter* phages, two *Citromicrobium* phages, and oceanic representative phages. **(A)** Heatmap displaying relative abundances of phages. **(B)** Box plots indicate the relative abundance of phages in upper-ocean samples (< 200 m) compared with their abundance in mesopelagic waters (200–1,000 m). The relative abundance is expressed by TPM (transcripts per million) values and described with log_10_ transformation.

The biogeographical distribution patterns of phages R34L1 and R34L2 were examined across the Global Ocean Viromes (GOV v2.0) data set. Both phages R34L1- and R34L2-type phage groups exhibited detectable signals in epipelagic (0–200 m) and mesopelagic (200–1,000 m) water, with varying relative abundances (Figure 6). Notably, neither genome was detected in viromes from the deep sea (> 1,000 m). The relative abundances of our phages and their related phages were consistently lower than those of the reference phages analyzed under the same methodology. Moreover, the distribution patterns of different erythrobacterial phages varied considerably, with R6L-type phage group being the most widespread, followed by L02-type phage group, and finally R34L1- and R34L2-type phage groups.

## 4 Discussion

Bacteriophages play a crucial role in marine ecosystems. They can regulate bacterial populations by infecting and killing bacteria, participate in nutrient cycling by releasing bacterial contents into the water, maintain ecosystem balance by interacting with bacteria, facilitate genetic exchange and evolution by horizontal gene transformation, and impact carbon cycling by breaking down bacteria (Brown et al., 2022; Focardi et al., 2020; Luo et al., 2017; Sanz-Gaitero et al., 2021). Despite their importance, research on phages targeting the genus *Erythrobacter* in the coastal euphotic zone remains significantly limited, with only two bacteriophage strains reported to date (Li et al., 2022; Lu et al., 2017). This study identified two novel phages infecting *Erythrobacter* named R34L1 and R34L2 (R34L2 being a sub-strain of R34L1), representing a new genus of *Eausmariqdvirus*.

The infection dynamics and environmental adaptability of phages R34L1 and R34L2, as evidenced by their latent periods, burst sizes, physical stability profiles, and genomic characteristics, offer critical insights into their ecological strategies and evolutionary trajectories. The latent periods of ~ 160 min observed in both phages are comparable to the latent period (<1 ~ 6 h) and burst size (27 ~ 1,500 PFU/cell) reported for the two *Erythrobacter* and most *Roseobacter*-infecting phages (Cai et al., 2019; Li et al., 2016; Zhao et al., 2009). Besides, the physicochemical stability profiles of R34L1 and R34L2 further suggested that phages R34L1 and R34L2 may have a broad distribution range and could potentially thrive in diverse environmental conditions.

The genomic G + C contents of R34L1 (61.60%) and R34L2 (61.19%) showed divergence from the other two *Erythrobacter* phages (59.43 and 66.52%) (Li et al., 2022; Lu et al., 2017), suggesting that they may have distinct evolutionary adaptations or selective pressures (Mann and Chen, 2010; Das and Rahlff, 2024). Among all 27 functionally related ORFs, eight ORFs are related to DNA replication and metabolism. Four ORFs are specifically involved in DNA synthesis, including DNA-directed DNA polymerase family A palm domain-containing protein (ORF65, R34L1), ssDNA-binding protein (ORF66), exonuclease (ORF67), and DNA primase/polymerase bifunctional N-terminal domain-containing protein (ORF72). The ssDNA-binding protein and exonuclease are involved in the proofreading and repair processes of DNA, by removing nucleotides from the ends of DNA strands and cleaning damaged DNA fragments to maintain genomic stability (Marceau, 2012). The N-terminal domain of bifunctional DNA primase-polymerase should play a crucial role in accommodating the DNA duplex by the prim-pol domain, regulating the selection of replication starting points and maintaining the stability of the replication process through its interaction with DNA polymerase (Guo et al., 2019; Lipps et al., 2004). The SAM-dependent methyltransferase (ORF64) catalyzes the transfer of methyl groups from S-adenosylmethionine (SAM) to a variety of acceptor substrates, related to proteins, DNA, and polysaccharide metabolites (Struck et al., 2012). And, SAM-dependent methyltransferase may also be involved in BacteRiophage Exclusion (BREX) defense mechanism in the host cells (Went et al., 2024). BREX is a defense system in prokaryotes and can act by providing restriction-modification (R-M) systems that epigenetically modify some specific sites (methylation) in the genome of the host, resulting in the restriction-cleavage of incoming phage DNA by endonucleases because of these modifications lacking (Isaev et al., 2020; Raleigh and Brooks, 1998). Thus, SAM-dependent methyltransferase may help phages evade degradation by host endonucleases. The ParB/Sulfiredoxin domain (ORF29) exhibits NTPase and DNase activities, which facilitate the separation and division of the host chromosome, thereby supporting phage replication (Maindola et al., 2014; Osorio-Valeriano et al., 2019). The DEAD/DEAH box helicase (ORF62) regulates ATP binding and hydrolysis and plays pivotal roles in viral RNA replication and transcription (Tanner et al., 2003; Tuteja and Tuteja, 2004). The VRR-NUC domain (ORF63), which belongs to an ancient restriction endonuclease-like superfamily, is closely involved in phage infection and gene expression regulation and repair, ensuring the stability and integrity of phage DNA during replication (Elahi et al., 2022; Pennell et al., 2014).

The glycoside hydrolase family 19 catalytic domain-containing protein (ORF33) has been predicted to be associated with host cell lysis (Orlando et al., 2021; Valero-Rello, 2019). The glycoside hydrolase family 19 (GH19 family), encompassing both chitinases and endolysins, is well-recognized for its dual functionality. This family has been extensively investigated for its potential applications in managing plant fungal infestations, facilitating the recycling of chitin-rich biomass, and combating bacteria that exhibit resistance to multiple drugs (Orlando et al., 2021). Based on the analysis by the Carbohydrate-Active EnZymes database (CAZy) (Cantarel et al., 2009), ORF33 is likely to be an endolysin (EC 3.2.1.17) rather than a chitinase (EC 3.2.1.14). In addition, the GH19 family is highly diverse and widely distributed across plants, fungi, bacteria, and phages, exhibiting notable geographical and ecological variability (Orlando et al., 2021). However, in the GH19-based tree (Figure 5A), our phages were found to cluster with bacterial sequences, such as *Erythrobacter*, *Sphingomonas*, *Caulobacter*, and *Phenylobacterium*, instead of with other phages. This unusual clustering suggests a potential horizontal gene transfer event, where the GH19 gene of phages R34L1 and R34L2 may have been acquired from a bacterial host, specifically *Erythrobacter*. Meanwhile, GH19 homologs were not detected in *Sphingomonas* phage Carli, *Burkholderia* phage BcepNazgul, indicating that GH19 sequences are not universally present or well-identified in phages. As previously reported, numerous phage GH19 sequences cluster with bacterial homologs, likely evolving from insertions under the selective pressure of the co-evolutionary phage-bacteria interaction process (Orlando et al., 2021). Here, we report the first identification of GH19 sequences in AAPB-isolated phages, highlighting the dynamic nature of phage genomes and their ability to acquire genes from their bacterial hosts.

AMGs are genes carried by phages that encode proteins involved in host metabolic pathways, and these genes are believed to have been acquired from their hosts or other phages by horizontal gene transfer (Lawrence et al., 2002). Studies have shown that AMGs can enhance viral replication and influence the metabolic processes of their hosts (Hurwitz and U’Ren, 2016; Thompson et al., 2011). In our study, GapR (ORF28) was identified for the first time in the phage genome. GapR is typically a chromosome structuring protein that plays a crucial role in bacterial DNA replication (Guo et al., 2018; Tarry et al., 2019). Specifically, GapR forms a dimer-of-dimers that fully encircles overtwisted DNA, stimulating gyrase and topo IV to relax positive supercoils caused by the DNA unwinding during replication (Guo et al., 2018). Interestingly, GapR homologs with high similarity to that of R34L1 have also been identified in the genomes of several *Erythrobacter* strains (Aguirre and Schwartzman, 2024; Chen et al., 2020; Probst et al., 2020), suggesting that our phages may have acquired the GapR gene during a specific infection event.

**Figure 7:**
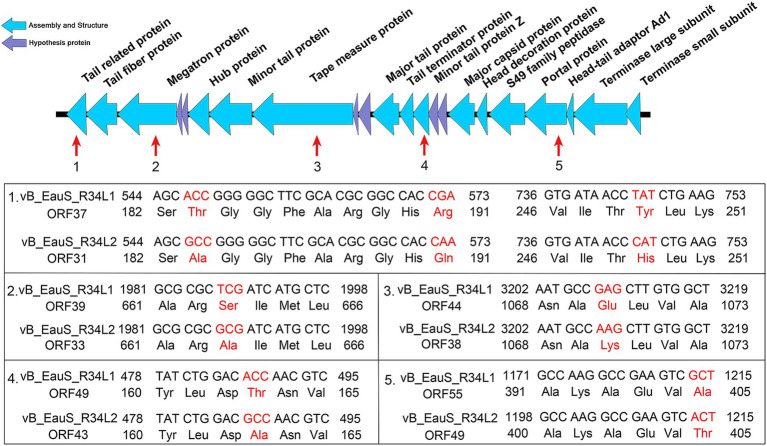
The point mutations of phage tail-related genes between phages vB_EauS-R34L1 and vB_EauS-R34L2.

Theoretically, variations in the host range of the tailed phages are frequently associated with the receptor-binding proteins (RBP) located at the distal end of the tail, including tail spikes, extended tail fibers, and central tail spike proteins, which are responsible for host recognition and adsorption (de Jonge et al., 2019; Nobrega et al., 2018; Yehl et al., 2019). In the amino acid sequence of all predicted function ORFs, seven point mutations were detected (Figure 7), including tail-related protein (ORF 37 of phage R34L1 and ORF 31 of phage R34L2), tip attachment protein J domain-containing protein (ORF 33 and ORF 39), phage tail length tape measure family protein (ORF 38 and ORF 44), minor tail protein Z (ORF 43 and ORF 49) and portal protein (ORF 49 and ORF 55). Additionally, two ORFs are likely associated with host adsorption: the tail fiber protein (ORF 32 and ORF 38) and the tip attachment protein J domain-containing protein (Ge and Wang, 2024). The amino acid sequence similarity between the tail fiber proteins of the two phages is 100%, while the similarity between the tip attachment protein J domain-containing proteins is 99.87%. Notably, the point mutation in tip attachment protein J domain-containing protein occurs in the C terminus (Figure 7), which is known to play a significant role in determining the host range (Yehl et al., 2019). Point mutations in RBPs have been shown to lead to host range expansion in phages, by altering their ability to recognize and bind to specific bacterial receptors (Subramanian et al., 2022). In our study, the tail fiber protein of phages R34L1 and R34L2 exhibited low sequence identity (48.18%) and query cover (26.51%) when compared to the adsorption-related protein (tail fiber protein, ORF 25) of phage L02. In contrast, no significant similarity was observed with the corresponding protein of phage R6L. These findings provide more evidence for the differences in host range and highlight the diversity among *Erythrobacter* phages. However, further investigation is needed to elucidate the factors determining the host ranges of *Erythrobacter* phages.

Strikingly, phages R34L1/R34L2 exhibit a remarkable evolutionary distinction from existing phages uploaded in public databases. Their average nucleotide identity (ANI) values with other phages fall below the 70% threshold (Figure 3C), a widely accepted benchmark for delineating novel genera in phage taxonomy (Turner et al., 2021). Moreover, their intergenomic similarity with other characterized phages is remarkably low (4.46–8.54%) (Figure 3D), reinforcing their taxonomic novelty. Additionally, comparative genomic analysis revealed low identities (23.86–60.76%) among the ORFs (33/78 for R34L1 and 30/72 for R34L2) that have homology to those of the two previously reported *Erythrobacter* phages. Phylogenetic analysis by VICTOR positioned these phages on a deeply rooted branch within the family *Casjensviridae* (Figure 4A), further indicating their novelty. Phylogenetic analysis of the three key genes (Figure 5) also reveals unique catalytic motifs and structural differences. Overall, all results consistently support classifying R34L1 and R34L2 as a new genus, named *Eausmariqdvirus*, within the family *Casjensviridae*, underscoring the untapped diversity in marine phage populations.

Metagenomic fragment recruitment analysis showed R34L1- and R34L2-type phage groups exhibit a relatively restricted global distribution, with them detected in only a limited number of viromes from the GOV database, spanning both epipelagic and mesopelagic waters (Figure 6). Similarly, the previous two *Erythrobacter* phages also exhibited comparable distribution characteristics, suggesting that *Erythrobacter* phages may have relatively broad distribution patterns in marine epipelagic zones, in agreement with the general distribution of *Erythrobacter* in the environment (Jiao et al., 2007; Kolber et al., 2001; Shiba and Simidu, 1982). This limited distribution may reflect their high host specificity or distinct environmental adaptation strategies. Notably, large differences in the global distribution trends of the four phages were identified, while the genomic compositions of these phages are quite different. This result may reflect differential responses among phages to host physiological states or localized environmental factors (e.g., light, nutrient availability, temperature). These findings underscore the ecological diversity of marine phages that even within a shared host system, divergent host interaction modes or environmental adaptation mechanisms may allow coexisting phages to occupy distinct inches, thereby maintaining viral community stability and functional redundancy.

## 5 Conclusion

As an important AAPB genus in the ocean, *Erythrobacter* plays a significant role in the oceanic carbon cycle. In this study, we isolated and characterized one novel *Erythrobacter* phages, R34L1, and its sub-strain R34L2, marking significant advancements in our understanding of this understudied phage group. Notably, we identified the GapR gene as an AMG within phage genomes for the first time and discovered a homolog of the GH19 family sequence in AAPB-isolated phages—a finding previously unreported in this context. Taxonomic and genomic analyses indicated that phages R34L1 and R34L2 share a distant evolutionary relationship with known phages and form a distinct viral genus cluster within the family *Casjensviridae*, for which we propose to name *Eausmariqdvirus*. Ecological distribution analysis revealed that R34L1 and R34L2-like phages exclusively prefer the temperate and tropical epipelagic zones of the ocean, suggesting niche-specific adaptations. Overall, this study provides more information on the poorly understood *Erythrobacter* phages and deepens our understanding of the phage-host interactions in complex environments.

## Data Availability

The datasets presented in this study can be found in online repositories. The names of the repository/repositories and accession number(s) can be found below: https://www.ncbi.nlm.nih.gov/genbank/, PQ394074; https://www.ncbi.nlm.nih.gov/genbank/, PQ394075.
