# Supplementary material for: Two novel phages infecting Erythrobacter isolated from the epipelagic ocean
Source: Front Microbiol. 2025 Jun 10;16:1592355. doi: 10.3389/fmicb.2025.1592355 (PMC12185513; doi:10.3389/fmicb.2025.1592355)
Supplement: Supplementary file 1 [file Supplementary_file_1.docx]

Supplementary Material

# Supplementary Figures and Tables

## Supplementary Figures


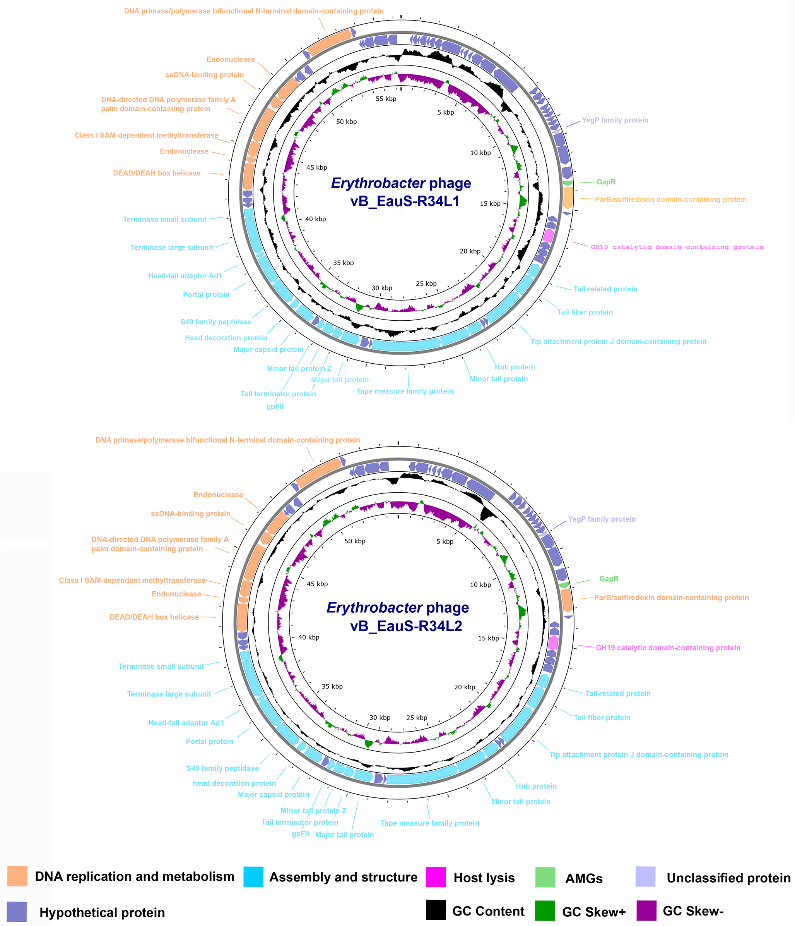


**Supplementary Figure S1.** Genomic structure of phage vB_EauSS-R34L1 and vB_EauSS-R34L2. The first and second outmost circles comprising arrows represent predicted ORFs. The third outmost circle represents the GC contents. The fourth outmost circle represents the GC-skewness values (GC-skew=(G-C)/(G +C)). The scale representing the genome size (bp) is displayed using the innermost circle.

## Supplementary Tables

**Supplementary Table S1.** Host range of the phage vB_EauS-R34L1 and vB_EauS-R34L2

| **Strains** | **Best matched species**  **(% Id of 16S rDNA)** | **Source and location** | **vB_EauS-R34L1** | **vB_EauS-R34L2** |
| --- | --- | --- | --- | --- |
| *Erythrobacter* sp. JL 475 | *E. aurantius* strain C5  (99.93%) | Surface sea water, South China Sea, China (Zheng et al., 2016) | + | + |
| JL 967 | *Erythrobacter* sp. M71_W20  (100.00) | Surface sea water, Taiwan strait, China | - | + |
| JL 2286 | *E. flavus* SW-46(T)  (100.00) | Surface water, Western Pacific Ocean | - | + |
| *E.* *litoralis* DSM 8509 |  | Cyanobacterial mat, Netherlands (Yurkov et al., 1994) | - | - |
| *E. longus* DSM 6997 |  | Seaweed *Enteromorpha linza*, Japan (Shiba et al., 1982) | - | - |
| *Erythrobacter* sp. NAP1 |  | Surface seawater, Northwestern Atlantic Ocean (Koblížek et al., 2011) | - | - |
| 1. *westpacificensis* JCM18014T |  | Surface water, Western Pacific Ocean (Wei et al., 2013) | - | - |
| JL 917 | *E. citreus* RE35F/1  (99.72) | Surface sea water, Taiwan strait, China | - | - |
| JL 1267 | *Erythrobacter* sp. MON004  (100.00) | Surface sea water, South China sea, China | - | - |
| JL 274-1 | *E. Vulgaris* 022 2-10  (99.22) | Changjiang Estuary, China | - | - |
| JL 658-2 | *E. citreus* RE35F/1  (99.66) | Surface sea water, Taiwan strait, China | - | - |
| JL 883 | *E. flavus* SW-46(T)  (99.79) | Surface sea water, Western Pacific Ocean | - | - |
| JL 971-1 | *E. nanhaisediminis* T30(T)  (99.33) | Surface sea water, Taiwan Strait, China | - | - |
| JL 1033 | *E. nanhaisediminis* T30(T)  (99.69) | 50 m sea water, Western Pacific Ocean | - | - |
| JL 1058 | *E. citreus* RE35F/1(T)  (98.52) | 150 m sea water, Western Pacific Ocean | - | - |
| JL 1059 | *E. nanhaisediminis* T30(T)  18(99.22) | 150 m sea water, East China Sea, China | - | - |
| JL 1201 | *E. Vulgaris* TVG01-C004  (99.80) | Surface sea water, West Pacific Ocean | - | - |
| JL 1408 | *E. flavus* SW-46(T)  (99.89) | Surface sea water, South China Sea, China | - | - |
| JL 1500 | *E. Pelagi* UST081027-248(T)  (99.90) | 60 m sea water, South China Sea, China | - | - |
| JL 1833 | *E. flavus* BL16  (100.00) | Bottom sea water, South China sea, China | - | - |
| JL 2316 | *Erythrobacter* sp. CC-AMZ-30L  (97.12) | Surface sea water, Pacific Ocean | - | - |
| JL 923 | *E. flavus* SW-46(T)  (99.25) | Surface sea water, South China Sea, China | - | - |
| JL 1302 | *E. Nanhaisediminis* T30  (97.79) | Surface sea water, South China Sea, China | - | - |
| JL 1463 | *E.* Pelagi UST081027-248  (98.48) | Surface sea water, South China Sea, China | - | - |
| JL 1317 | *E. flavus* SW-46(T)  (99.01) | Surface sea water, South China Sea, China | - | - |
| JL 3201 | *E. longus* DSM 6997(T)  (98.43) | Surface sea water, South China Sea, China | - | - |
| JL 3203 | *E. longus* DSM 6997(T)  (99.13) | Surface sea water, South China Sea, China | - | - |
| JL 3235 | *E. longus* DSM 6997(T)  (99.00) | Surface sea water, South China Sea, China | - | - |
| JL 3238 | *E. longus* DSM 6997(T)  (99.28) | Surface sea water, South China Sea, China | - | - |
| JL 3245 | *E. longus* DSM 6997(T)  (99.35) | Surface sea water, South China Sea, China | - | - |
| JL 3583 | *E. longus* DSM 6997(T)  (99.28) | Surface sea water, South China Sea, China | - | - |
| *Dinoroseobacter shibae* DFL12^T^ |  | Cells of *Prorocentrum lima* (Swingley et al., 2007) | - | - |
| *Roseobacter lineage* DSM7001 |  | Seaweed, Japan (Biebl et al., 2005) | - | - |
| *C. bathyomarinum* JL354 |  | Surface sea water, South China sea, China (Jiao et al., 2010) | - | - |
| JL31 | *C. bathyomarinum* JF-1(T)  (99.42) | Surface sea water, South China Sea, China | - | - |
| JL 269 | *C. bathyomarinum* JF-1(T)  (98.99) | Surface sea water, East China Sea, China | - | - |
| JL 329 | *C. bathyomarinum* JF-1(T)  (98.97) | Surface sea water, South China Sea, China | - | - |
| JL 346 | *C. bathyomarinum* JF-1(T)  (97.25) | Surface sea water, South China Sea, China | - | - |
| JL 477 | *C. bathyomarinum* JF-1(T)  (99.41) | Surface sea water, South China Sea, China | - | - |
| JL 522-1 | *C. bathyomarinum* JF-1(T)  (99.55) | Surface sea water, South China Sea, China | - | - |
| JL 1010 | *C. bathyomarinum* JF-1(T)  (99.51) | Surface sea water, East China Sea, China | - | - |
| JL 1035 | *Citromicrobium*  (98.92) | 50 m sea water, East China Sea, China | - | - |
| JL 1039 | *C. bathyomarinum* JF-1(T)  (99.28) | 50 m sea water, East China Sea, China | - | - |
| JL 1197 | *C. bathyomarinum* JF-1(T)  (99.38) | 50 m sea water, East China Sea, China | - | - |
| JL 1351 | *C. bathyomarinum* JF-1(T)  (99.36) | Surface sea water, South China Sea, China | - | - |
| JL 1366 | *C. bathyomarinum* JF-1(T)  (99.36) | Surface sea water, South China Sea, China | - | + |
| JL 1455 | *C. bathyomarinum* JF-1(T)  (97.86) | 17 m sea water, South China Sea, China | - | - |
| JL 2201 | *C. bathyomarinum* JF-1(T)  (99.16) | Surface water, Eastern Atlantic Ocean | - | - |
| JL 2308 | *C. bathyomarinum* JF-1(T)  (95.75) | Surface sea water, Pacific Ocean | - | - |
| WPS32 | *C. bathyomarinum* JF-1(T)  (99.77) | Surface sea water, South Pacific Ocean | - | - |

**Supplementary Table S2.** Genome annotation of phage vB_EauS-R34L1

| **ORF** | **Start** | **Stop** | **Strand** | **Annotation** | **Database** | **Best-matched evidence or organism** | **Accession Num.** | **%.idt** | **evalue** |
| --- | --- | --- | --- | --- | --- | --- | --- | --- | --- |
| 1 | 496 | 672 | - |  |  |  |  |  |  |
| 2 | 669 | 929 | - |  |  |  |  |  |  |
| 3 | 926 | 1078 | - | Hypothetical protein | nr | *Sphingopyxis* sp. | NJS14063 | 71.88 | 8.00E-07 |
| 4 | 1075 | 1473 | - |  |  |  |  |  |  |
| 5 | 1470 | 1658 | - |  |  |  |  |  |  |
| 6 | 1655 | 1975 | - |  |  |  |  |  |  |
| 7 | 1975 | 2370 | - |  |  |  |  |  |  |
| 8 | 2367 | 3542 | - |  |  |  |  |  |  |
| 9 | 3580 | 3702 | - |  |  |  |  |  |  |
| 10 | 3766 | 4005 | - |  |  |  |  |  |  |
| 11 | 4069 | 4272 | - |  |  |  |  |  |  |
| 12 | 4302 | 4910 | - | Hypothetical protein | TrEMBL | ECO:0000313\|EMBL:MAK72262.1 | A0A2D7YS48 | 56.63 | 6.01E-43 |
| 13 | 4897 | 5808 | - | Hypothetical protein | TrEMBL | ECO:0000313\|EMBL:MAK72263.1 | A0A2D7YS88 | 76.24 | 1.97E-131 |
| 14 | 5865 | 7535 | - | Hypothetical protein | TrEMBL | ECO:0000313\|EMBL:MAK72264.1 | A0A2D7YS49 | 57.71 | 4.27E-165 |
| 15 | 8141 | 8416 | + | Hypothetical protein | TrEMBL | ECO:0000313\|EMBL:MAK72265.1 | A0A2D7YS58 | 72.53 | 7.60E-28 |
| 16 | 8446 | 8820 | + | Hypothetical protein | nr | *Idiomarinaceae* bacterium | MAK72266 | 46.00 | 8.00E-12 |
| 17 | 8817 | 9083 | + |  |  |  |  |  |  |
| 18 | 9183 | 9431 | + | Hypothetical protein | TrEMBL | ECO:0000313\|EMBL:TXH41226.1 | A0A5C7P5Y3 | 53.95 | 2.20E-14 |
| 19 | 9428 | 9643 | + |  |  |  |  |  |  |
| 20 | 9640 | 9903 | + | Hypothetical protein | TrEMBL | {ECO:0000313\|EMBL:MAK71935.1 | A0A2D7YR83 | 55.84 | 4.84E-14 |
| 21 | 9912 | 10118 | + |  |  |  |  |  |  |
| 22 | 10115 | 10366 | + | Hypothetical protein | TrEMBL | ECO:0000313\|EMBL:MAK71936.1 | A0A2D7YR80 | 60.24 | 1.02E-19 |
| 23 | 10363 | 10668 | + | Hypothetical protein | RefSeq | *Rhizorhabdus wittichii* | WP_208631604 | 35.56 | 3.95E-08 |
| 24 | 10670 | 10864 | + | YegP family protein | nr | *Clostridia* *bacterium* | MBQ4150558 | 61.90 | 6.00E-11 |
| 25 | 10857 | 11579 | + | Hypothetical protein | TrEMBL | ECO:0000313\|EMBL:MAK71938.1 | A0A2D7YR91 | 61.28 | 7.17E-81 |
| 26 | 11576 | 12577 | + | Hypothetical protein | TrEMBL | ECO:0000313\|EMBL:MAK71940.1 | A0A2D7YRC0 | 66.67 | 1.76E-118 |
| 27 | 12717 | 13376 | + | Hypothetical protein | TrEMBL | ECO:0000313\|EMBL:MAK71941.1 | A0A2D7YR89 | 61.50 | 2.67E-67 |
| 28^1^ | 13454 | 13753 | + | GapR |  | *Caulobacter cresentus* |  |  |  |
| 29 | 13783 | 14985 | + | ParB/Sulfiredoxin domain-containing protein | TrEMBL | ECO:0000256\|Google:ProtNLM | A0A2D7YR92 | 69.70 | 1.27E-143 |
| 30 | 15148 | 15321 | + |  |  |  |  |  |  |
| 31 | 15433 | 15762 | - |  |  |  |  |  |  |
| 32 | 15765 | 16196 | - |  |  |  |  |  |  |
| 33 | 16207 | 16995 | - | Glycoside hydrolase family 19 catalytic domain-containing protein | TrEMBL | ECO:0000256\|Google:ProtNLM | A0A5C7P5Z3 | 59.62 | 6.55E-84 |
| 34 | 16998 | 17411 | - | Hypothetical protein | gpu | *Sphingomonas* phage Carli | OR225223 | 36.64 | 2.38E-13 |
| 35 | 17389 | 17829 | - |  |  |  |  |  |  |
| 36 | 17784 | 18290 | - | Hypothetical protein | RefSeq | uncultured *Sphingomonas* sp. | WP_294392014 | 49.66 | 1.02E-33 |
| 37 | 18408 | 19181 | - | Tail-related protein | nr | *Citromicrobium* phage vB_CbaS-RXM | USM11576 | 34.04 | 2.00E-24 |
| 38^1^ | 19178 | 20425 | - | Tail fiber protein |  | *Dinoroseobacter* phage vB_DshS-R4C |  |  |  |
| 39 | 20454 | 22817 | - | Tip attachment protein J domain-containing protein | TrEMBL | ECO:0000256\|Google:ProtNLM | A0A5C7P1U6 | 49.29 | 3.29E-204 |
| 40 | 22807 | 23016 | - | Hypothetical protein | RefSeq | *Thermomonas* sp. S9 | WP_259304481 | 51.61 | 8.08E-09 |
| 41 | 23013 | 23246 | - | Hypothetical protein | TrEMBL | ECO:0000313\|EMBL:TXH41245.1 | A0A5C7P4E8 | 61.33 | 6.45E-19 |
| 42^1^ | 23258 | 24094 | - | Hub protein |  | *Dinoroseobacter* phage vB_DshS-R4C |  |  |  |
| 43 | 24091 | 25833 | - | Minor tail protein | TrEMBL | ECO:0000256\|Google:ProtNLM | A0A5C7P1J5 | 40.67 | 3.01E-84 |
| 44 | 25833 | 29885 | - | Tape measure family protein | RefSeq | uncultured *Sulfitobacter* sp. | WP_288959817 | 32.16 | 1.21E-175 |
| 45 | 29878 | 30060 | - |  |  |  |  |  |  |
| 46 | 30084 | 30563 | - | Hypothetical protein | gpu | *Aeromonas* phage phiWae15 | LC778250 | 32.43 | 2.71E-09 |
| 47 | 30644 | 31720 | - | Hypothetical protein | TrEMBL | ECO:0000313\|EMBL:TXH56978.1 | A0A5C7QE11 | 60.77 | 1.12E-116 |
| 48 | 31745 | 32281 | - | Tail terminator | nr | *Serratia* phage KpYy 1 41 | YP_010161342 | 40.48 | 7.00E-22 |
| 49 | 32278 | 32901 | - | Minor tail protein Z | nr | *Caudoviricetes* sp. | DAG19488 | 51.01 | 1.00E-58 |
| 50 | 32898 | 33266 | - | Hypothetical protein | TrEMBL | ECO:0000313\|EMBL:TXH56981.1 | A0A5C7QE70 | 51.69 | 3.83E-27 |
| 51 | 33266 | 33622 | - | Hypothetical protein | TrEMBL | ECO:0000313\|EMBL:TXH56982.1 | A0A5C7QD92 | 45.45 | 6.88E-05 |
| 52 | 33691 | 34746 | - | Major capsid protein | RefSeq | uncultured *Sulfitobacter* sp. | WP_288959824 | 68.01 | 1.09E-137 |
| 53 | 34821 | 35234 | - | Head decoration protein | RefSeq | uncultured *Sulfitobacter* sp. | WP_288959825 | 46.67 | 8.17E-22 |
| 54 | 35322 | 36761 | - | S49 family peptidase | TrEMBL | ECO:0000313\|EMBL:TXH56985.1 | A0A5C7QF16 | 45.02 | 1.30E-95 |
| 55 | 36748 | 38433 | - | Portal protein | TrEMBL | ECO:0000313\|EMBL:TXH57010.1 | A0A5C7QCX6 | 61.10 | 5.25E-187 |
| 56 | 38433 | 38690 | - | Head-tail adaptor Ad1 | RefSeq | *Klebsiella* phage KPN N137 | YP_009834130 | 43.75 | 4.41E-07 |
| 57 | 38697 | 40865 | - | Terminase large subunit | nr | *Pectobacterium* phage MA11 | YP_010000197 | 51.51 | 0 |
| 58 | 40807 | 41388 | - | Terminase small subunit | nr | *Burkholderia* phage BcepNazgul | NP_918999 | 39.74 | 7.00E-38 |
| 59 | 41436 | 41690 | - | Hypothetical protein | TrEMBL | ECO:0000313\|EMBL:TXH56989.1 | A0A5C7QDB9 | 58.06 | 3.86E-11 |
| 60 | 41659 | 42015 | - |  |  |  |  |  |  |
| 61 | 42028 | 42441 | - |  |  |  |  |  |  |
| 62 | 42438 | 44039 | - | DEAD/DEAH box helicase | TrEMBL | ECO:0000313\|EMBL:TXH56993.1 | A0A5C7QDA0 | 57.54 | 2.29E-160 |
| 63 | 44036 | 44401 | - | Endonuclease | nr | *Proteus* phage Saba | YP_009997885 | 43.48 | 5.00E-06 |
| 64 | 44394 | 45338 | - | Class I SAM-dependent methyltransferase | TrEMBL | ECO:0000313\|EMBL:TXH56997.1 | A0A5C7QDM6 | 66.34 | 5.26E-114 |
| 65 | 45340 | 47439 | - | DNA-directed DNA polymerase family A palm domain-containing protein | TrEMBL | ECO:0000259\|Pfam:PF00476 | A0A2D7YRJ0 | 84.35 | 3.16E-288 |
| 66 | 47552 | 48217 | - | ssDNA-binding protein | nr | uncultured *Sulfitobacter* sp. | WP_288959835 | 51.85 | 2.00E-56 |
| 67 | 48219 | 49652 | - | Exonuclease | nr | *Aeromonas* phage LAh_7 | YP_009998313 | 36.03 | 3.00E-72 |
| 68 | 49664 | 49765 | - |  |  |  |  |  |  |
| 69 | 49776 | 50207 | - | Hypothetical protein | TrEMBL | ECO:0000313\|EMBL:TXH57001.1 | A0A5C7QE90 | 42.64 | 2.17E-22 |
| 70 | 50357 | 50770 | - | Hypothetical protein | RefSeq | uncultured *Sulfitobacter* sp. | WP_288959838 | 37.82 | 2.47E-10 |
| 71 | 50914 | 51213 | + | Hypothetical protein | RefSeq | uncultured *Sulfitobacter* sp. | WP_288959839 | 37.78 | 1.46E-10 |
| 72 | 51216 | 53732 | + | DNA primase/polymerase bifunctional N-terminal domain-containing protein | TrEMBL | ECO:0000256\|Google:ProtNLM | A0A5C7QDB4 | 54.73 | 4.70E-265 |
| 73 | 53729 | 53974 | + |  |  |  |  |  |  |
| 74 | 53971 | 54069 | - |  |  |  |  |  |  |
| 75 | 54066 | 54251 | - |  |  |  |  |  |  |
| 76 | 54235 | 54798 | - | Hypothetical protein | RefSeq | *Mesorhizobium* sp. M2A.F.Ca.ET.039.01.1.1 | WP_127409445 | 41.76 | 4.70E-25 |
| 77 | 54795 | 55625 | - | Hypothetical protein | RefSeq | *Pseudomonas* | WP_198862599 | 29.03 | 7.03E-10 |
| 78 | 55618 | 56172 | - |  |  |  |  |  |  |

^1^ Predicted based on viral protein identification by mass spectrometry (unpublished data).

**Supplementary Table S3.** Genome annotation of phage vB_EauS-R34L2

| **ORF** | **Start** | **Stop** | **Strand** | **Annotation** | **Database** | **Best-matched evidence or organism** | **Accession Num.** | **%.idt** | **evalue** |
| --- | --- | --- | --- | --- | --- | --- | --- | --- | --- |
| 1 | 573 | 932 | - |  |  |  |  |  |  |
| 2 | 929 | 1663 | - |  |  |  |  |  |  |
| 3 | 1701 | 1823 | - |  |  |  |  |  |  |
| 4 | 1887 | 2126 | - |  |  |  |  |  |  |
| 5 | 2190 | 2393 | - |  |  |  |  |  |  |
| 6 | 2423 | 3031 | - | Hypothetical protein | TrEMBL | ECO:0000313\|EMBL:MAK72262.1 | A0A2D7YS48 | 56.02 | 3.90E-42 |
| 7 | 3018 | 3929 | - | Hypothetical protein | TrEMBL | ECO:0000313\|EMBL:MAK72263.1 | A0A2D7YS88 | 76.24 | 2.00E-131 |
| 8 | 3986 | 5656 | - | Hypothetical protein | TrEMBL | ECO:0000313\|EMBL:MAK72264.1 | A0A2D7YS49 | 57.71 | 4.30E-165 |
| 9 | 6357 | 6632 | + | Hypothetical protein | TrEMBL | ECO:0000313\|EMBL:MAK72265.1 | A0A2D7YS58 | 72.53 | 7.60E-28 |
| 10 | 6662 | 7036 | + | Hypothetical protein | TrEMBL | ECO:0000313\|EMBL:SNT20292.1 | A0A239KRC4 | 39.19 | 1.56E-04 |
| 11 | 7033 | 7299 | + |  |  |  |  |  |  |
| 12 | 7399 | 7647 | + | Hypothetical protein | TrEMBL | ECO:0000313\|EMBL:TXH41226.1 | A0A5C7P5Y3 | 53.95 | 2.20E-14 |
| 13 | 7644 | 7859 | + |  |  |  |  |  |  |
| 14 | 7856 | 8119 | + | Hypothetical protein | TrEMBL | ECO:0000313\|EMBL:MAK71935.1 | A0A2D7YR83 | 55.84 | 4.84E-14 |
| 15 | 8116 | 8334 | + |  |  |  |  |  |  |
| 16 | 8331 | 8582 | + | Hypothetical protein | TrEMBL | ECO:0000313\|EMBL:MAK71936.1 | A0A2D7YR80 | 60.24 | 1.02E-19 |
| 17 | 8579 | 8884 | + | Hypothetical protein | RefSeq | *Rhizorhabdus wittichii* | WP_208631604 | 36.67 | 1.36E-08 |
| 18 | 8886 | 9080 | + | YegP family protein | RefSeq | *Clostridia bacterium* | MBQ4150558 | 61.90 | 6.00E-11 |
| 19 | 9073 | 9795 | + | Hypothetical protein | TrEMBL | ECO:0000313\|EMBL:MAK71938.1 | A0A2D7YR91 | 61.28 | 7.17E-81 |
| 20 | 9792 | 10793 | + | Hypothetical protein | TrEMBL | ECO:0000313\|EMBL:MAK71940.1 | A0A2D7YRC0 | 66.67 | 1.76E-118 |
| 21 | 10933 | 11592 | + | Hypothetical protein | TrEMBL | ECO:0000313\|EMBL:MAK71941.1 | A0A2D7YR89 | 61.50 | 2.67E-67 |
| 22^1^ | 11670 | 11969 | + | GapR |  | *Caulobacter cresentus* |  |  |  |
| 23 | 11999 | 13201 | + | ParB/Sulfiredoxin domain-containing protein | TrEMBL | ECO:0000256\|Google:ProtNLM | A0A2D7YR92 | 69.70 | 1.27E-143 |
| 24 | 13364 | 13537 | + |  |  |  |  |  |  |
| 25 | 13649 | 13978 | - |  |  |  |  |  |  |
| 26 | 13981 | 14412 | - |  |  |  |  |  |  |
| 27 | 14423 | 15211 | - | Glycoside hydrolase family 19 catalytic domain-containing protein | TrEMBL | ECO:0000256\|Google:ProtNLM | A0A5C7P5Z3 | 59.62 | 6.55E-84 |
| 28 | 15214 | 15627 | - | Hypothetical protein | gpu | *Sphingomonas* phage Carli | OR225223 | 36.64 | 2.38E-13 |
| 29 | 15605 | 16045 | - |  |  |  |  |  |  |
| 30 | 16000 | 16506 | - | Hypothetical protein | RefSeq | uncultured *Sphingomonas* sp. | WP_294392014 | 49.66 | 1.02E-33 |
| 31 | 16624 | 17397 | - | Tail-related protein | nr | *Citromicrobium* phage vB_CbaS-RXM | USM11576 | 33.69 | 7.00E-24 |
| 32^1^ | 17394 | 18641 | - | Tail fiber protein |  | *Dinoroseobacter* phage vB_DshS-R4C |  |  |  |
| 33 | 18670 | 21033 | - | Tip attachment protein J domain-containing protein | TrEMBL | ECO:0000256\|Google:ProtNLM | A0A5C7P1U6 | 49.29 | 4.29E-204 |
| 34 | 21023 | 21232 | - | Hypothetical protein | RefSeq | *Thermomonas* sp. S9 | WP_259304481 | 51.61 | 8.08E-09 |
| 35 | 21229 | 21462 | - | Hypothetical protein | TrEMBL | ECO:0000313\|EMBL:TXH41245.1 | A0A5C7P4E8 | 61.33 | 6.45E-19 |
| 36^1^ | 21474 | 22310 | - | Hub protein |  | *Dinoroseobacter* phage vB_DshS-R4C |  |  |  |
| 37 | 22307 | 24049 | - | Minor tail protein | TrEMBL | ECO:0000256\|Google:ProtNLM | A0A5C7P1J5 | 40.67 | 3.01E-84 |
| 38 | 24049 | 28101 | - | Tape measure family protein | RefSeq | uncultured *Sulfitobacter* sp. | WP_288959817 | 32.08 | 3.51E-175 |
| 39 | 28094 | 28276 | - |  |  |  |  |  |  |
| 40 | 28300 | 28779 | - | Hypothetical protein | gpu | *Aeromonas* phage phiWae15 | LC778250 | 32.43 | 2.71E-09 |
| 41 | 28860 | 29936 | - | Major tail protein with Ig-like domain | nr | *Pectobacterium* phage MA11 | YP_010000206 | 39.15 | 2.00E-32 |
| 42 | 29961 | 30497 | - | Tail terminator | nr | *Serratia* phage KpYy 1 41 | YP_010161342 | 40.48 | 7.00E-22 |
| 43 | 30494 | 31117 | - | Minor tail protein Z | nr | *Caudoviricetes* sp. | DAG19488 | 51.01 | 6.00E-59 |
| 44^1^ | 31114 | 31482 | - | Stopper protein |  |  |  |  |  |
| 45 | 31482 | 31838 | - | Hypothetical protein | TrEMBL | ECO:0000313\|EMBL:TXH56982.1 | A0A5C7QD92 | 45.45 | 6.88E-05 |
| 46 | 31907 | 32962 | - | Major capsid protein | RefSeq | uncultured *Sulfitobacter* sp. | WP_288959824 | 68.01 | 1.09E-137 |
| 47 | 33037 | 33450 | - | Head decoration protein | RefSeq | uncultured *Sulfitobacter* sp. | WP_288959825 | 46.67 | 8.17E-22 |
| 48 | 33538 | 34977 | - | S49 family peptidase | TrEMBL | ECO:0000313\|EMBL:TXH56985.1 | A0A5C7QF16 | 45.02 | 1.30E-95 |
| 49 | 34964 | 36649 | - | Portal protein | TrEMBL | ECO:0000313\|EMBL:TXH57010.1 | A0A5C7QCX6 | 60.92 | 1.53E-186 |
| 50 | 36649 | 36906 | - | Head-tail adaptor Ad1 | RefSeq | *Klebsiella* phage KPN N137 | YP_009834130 | 43.75 | 4.41E-07 |
| 51 | 36913 | 39081 | - | Terminase large subunit | nr | *Pectobacterium* phage MA11 | YP_010000197 | 51.51 | 0.00 |
| 52 | 39023 | 39604 | - | Terminase small subunit | nr | *Burkholderia* phage BcepNazgul | NP_918999 | 39.74 | 7.00E-38 |
| 53 | 39652 | 39906 | - | Hypothetical protein | TrEMBL | ECO:0000313\|EMBL:TXH56989.1 | A0A5C7QDB9 | 58.06 | 3.86E-11 |
| 54 | 39875 | 40231 | - |  |  |  |  |  |  |
| 55 | 40244 | 40657 | - |  |  |  |  |  |  |
| 56 | 40654 | 42255 | - | DEAD/DEAH box helicase | TrEMBL | ECO:0000313\|EMBL:TXH56993.1 | A0A5C7QDA0 | 57.54 | 2.29E-160 |
| 57 | 42252 | 42617 | - | Endonuclease | nr | *Proteus* phage Saba | YP_009997885 | 43.48 | 5.00E-06 |
| 58 | 42610 | 43554 | - | Class I SAM-dependent methyltransferase | TrEMBL | ECO:0000313\|EMBL:TXH56997.1 | A0A5C7QDM6 | 66.34 | 5.3E-114 |
| 59 | 43556 | 45655 | - | DNA-directed DNA polymerase family A palm domain-containing protein | TrEMBL | ECO:0000259\|Pfam:PF00476 | A0A2D7YRJ0 | 84.35 | 3.16E-288 |
| 60 | 45768 | 46433 | - | ssDNA-binding protein | nr | uncultured *Sulfitobacter* sp. | WP_288959835 | 51.85 | 1.00E-56 |
| 61 | 46435 | 47868 | - | Exonuclease | nr | *Aeromonas* phage LAh_7 | YP_009998313 | 36.03 | 2.00E-72 |
| 62 | 47880 | 47981 | - |  |  |  |  |  |  |
| 63 | 47992 | 48423 | - | Hypothetical protein | TrEMBL | ECO:0000313\|EMBL:TXH57001.1 | A0A5C7QE90 | 42.64 | 1.66E-22 |
| 64 | 48573 | 48986 | - | Hypothetical protein | RefSeq | uncultured *Sulfitobacter* sp. | WP_288959838 | 36.97 | 5.51E-10 |
| 65 | 49130 | 49429 | + | Hypothetical protein | RefSeq | uncultured *Sulfitobacter* sp. | WP_288959839 | 37.78 | 1.46E-10 |
| 66 | 49432 | 51948 | + | DNA primase/polymerase bifunctional N-terminal domain-containing protein | TrEMBL | ECO:0000256\|Google:ProtNLM | A0A5C7QDB4 | 54.61 | 2.33E-264 |
| 67 | 51945 | 52190 | + |  |  |  |  |  |  |
| 68 | 52187 | 52285 | - |  |  |  |  |  |  |
| 69 | 52282 | 52467 | - |  |  |  |  |  |  |
| 70 | 52451 | 53014 | - | Hypothetical protein | RefSeq | *Mesorhizobium* sp. M2A.F.Ca.ET.039.01.1.1 | WP_127409445 | 41.76 | 4.7E-25 |
| 71 | 53011 | 53841 | - | Hypothetical protein | RefSeq | *Pseudomonas* | WP_198862599 | 29.03 | 7.03E-10 |
| 72 | 53834 | 54388 | - |  |  |  |  |  |  |

^1^ Predicted based on viral protein identification by mass spectrometry (unpublished data)

**Supplementary Table S4.** ORF similarity comparison between Phages vB_EauS-R34L1 and vB_EauS-R34L2

| **vB_EauS-R34L1**  **ORF ID** | **Annotation** | **vB_EauS-R34L2**  **ORF ID** | **Annotation** | **Similarity (%)** |
| --- | --- | --- | --- | --- |
| 1 |  |  |  |  |
| 2 |  | 1 |  | 95.18 |
| 3 | Hypothetical protein |  |  |  |
| 4 |  |  |  |  |
| 5 |  |  |  |  |
| 6 |  |  |  |  |
| 7 |  |  |  |  |
| 8 |  | 2 |  |  |
| 9 |  | 3 |  | 100.00 |
| 10 |  | 4 |  | 100.00 |
| 11 |  | 5 |  | 100.00 |
| 12 | Hypothetical protein | 6 | Hypothetical protein | 100.00 |
| 13 | Hypothetical protein | 7 | Hypothetical protein | 96.02 |
| 14 | Hypothetical protein | 8 | Hypothetical protein | 100.00 |
| 15 | Hypothetical protein | 9 | Hypothetical protein | 100.00 |
| 16 | Hypothetical protein | 10 | Hypothetical protein | 100.00 |
| 17 |  | 11 |  | 100.00 |
| 18 | Hypothetical protein | 12 | Hypothetical protein | 100.00 |
| 19 |  | 13 |  | 100.00 |
| 20 | Hypothetical protein | 14 | Hypothetical protein | 100.00 |
| 21 |  | 15 |  | 100.00 |
| 22 | Hypothetical protein | 16 | Hypothetical protein | 100.00 |
| 23 | Hypothetical protein | 17 | Hypothetical protein | 100.00 |
| 24 | YegP family protein | 18 | YegP family protein | 99.01 |
| 25 | Hypothetical protein | 19 | Hypothetical protein | 100.00 |
| 26 | Hypothetical protein | 20 | Hypothetical protein | 100.00 |
| 27 | Hypothetical protein | 21 | Hypothetical protein | 100.00 |
| 28 | DUF2312 domain-containing protein | 22 | DUF2312 domain-containing protein | 100.00 |
| 29 | ParB/Sulfiredoxin domain-containing protein | 23 | ParB/Sulfiredoxin domain-containing protei | 100.00 |
| 30 |  | 24 |  | 100.00 |
|  |  | 25 |  | 100.00 |
| 32 |  | 26 |  | 100.00 |
| 33 | Glycoside hydrolase family 19 catalytic domain-containing protein | 27 | Glycoside hydrolase family 19 catalytic domain-containing protein | 100.00 |
| 34 | Hypothetical protein | 28 | Hypothetical protein | 100.00 |
|  |  |  |  | 100.00 |
| 36 | Hypothetical protein | 30 | Hypothetical protein |  |
| 37 | Tail-related protein | 31 | Tail-related protein | 100.00 |
| 38 | DUF2793 domain-containing protein | 32 | DUF2793 domain-containing protein | 98.83 |
| 39 | Tip attachment protein J domain-containing protein | 33 | Tip attachment protein J domain-containing protein | 100.00 |
| 40 | Hypothetical protein | 34 | Hypothetical protein | 99.87 |
| 41 | Hypothetical protein | 35 | Hypothetical protein | 100.00 |
| 42 | DUF2163 domain-containing protein | 36 | DUF2163 domain-containing protein | 100.00 |
| 43 | Minor tail protein | 37 | Minor tail protein | 100.00 |
| 44 | Phage tail length tape measure family protein | 38 | Phage tail length tape measure family protein | 100.00 |
| 45 |  | 39 |  | 99.93 |
| 46 | Hypothetical protein | 40 | Hypothetical protein | 100.00 |
| 47 | Major tail protein | 41 | Major tail protein | 100.00 |
| 48 | Tail terminator | 42 | Tail terminator | 100.00 |
| 49 | Minor tail protein | 43 | Minor tail protein | 100.00 |
| 50 | Hypothetical protein | 44 | Hypothetical protein | 99.52 |
| 51 | Hypothetical protein | 45 | Hypothetical protein | 100.00 |
| 52 | Major capsid protein | 46 | Major capsid protein | 100.00 |
| 53 | Head decoration protein | 47 | Head decoration protein | 100.00 |
| 54 | S49 family peptidase | 48 | S49 family peptidase | 100.00 |
| 55 | Portal protein | 49 | Portal protein | 100.00 |
| 56 | Head-tail adaptor Ad1 | 50 | Head-tail adaptor Ad1 | 99.82 |
| 57 | Terminase large subunit | 51 | Terminase large subunit | 100.00 |
| 58 | Terminase small subunit | 52 | Terminase small subunit | 100.00 |
| 59 | Hypothetical protein | 53 | Hypothetical protein | 100.00 |
| 60 |  | 54 |  | 100.00 |
| 61 |  | 55 |  | 100.00 |
| 62 | DEAD/DEAH box helicase | 56 | DEAD/DEAH box helicase | 100.00 |
| 63 | Endonuclease | 57 | Endonuclease | 100.00 |
| 64 | Class I SAM-dependent methyltransferase | 58 | Class I SAM-dependent methyltransferase | 100.00 |
| 65 | DNA-directed DNA polymerase family A palm domain-containing protein | 59 | DNA-directed DNA polymerase family A palm domain-containing protein | 100.00 |
| 66 | ssDNA-binding protein | 60 | ssDNA-binding protein | 100.00 |
| 67 | Exonuclease | 61 | Exonuclease | 99.10 |
| 68 |  | 62 |  | 99.79 |
| 69 | Hypothetical protein | 63 | Hypothetical protein | 96.97 |
| 70 | Hypothetical protein | 64 | Hypothetical protein | 97.90 |
| 71 | Hypothetical protein | 65 | Hypothetical protein | 98.54 |
| 72 | DNA primase/polymerase bifunctional N-terminal domain-containing protein | 66 | DNA primase/polymerase bifunctional N-terminal domain-containing protein | 100.00 |
| 73 |  | 67 |  | 99.64 |
| 74 |  | 68 |  | 100.00 |
| 75 |  | 69 |  | 100.00 |
| 76 | Hypothetical protein | 70 | Hypothetical protein | 100.00 |
| 77 | Hypothetical protein | 71 | Hypothetical protein | 100.00 |
| 78 |  | 72 |  | 94.57 |

**References**

Biebl, H., Allgaier, M., Tindall, B.J., Koblizek, M., Lünsdorf, H., Pukall, R., et al. (2005) *Dinoroseobacter shibae* gen. nov., sp. nov., a new aerobic phototrophic bacterium isolated from dinoflagellates. *Int. J. Syst. Evol. Microbiol.* 55, 1089-1096. doi:10.1099/ijs.0.63511-0

Jiao, N., Zhang, R., and Zheng, Q. (2010) Coexistence of Two Different Photosynthetic Operons in *Citromicrobium bathyomarinum* JL354 As Revealed by Whole-Genome Sequencing. *J. Bacteriol.* 192, 1169-1170. doi:10.1128/JB.01504-09

Koblížek, M., Janouškovec, J., Oborník, M., Johnson, J.H., Ferriera, S., and Falkowski, P.G. (2011) Genome sequence of the marine photoheterotrophic bacterium *Erythrobacter* sp. strain NAP1. *J. Bacteriol.* 193. doi:10.1128/jb.05845-11

Shiba, T., and Simidu, U. (1982) *Erythrobacter longus* gen. Nov., sp. nov., an aerobic bacterium which contains bacteriochlorophyll *a*. *Int. J. Syst. Bacteriol.* 32, 211-217. doi:10.1099/00207713-32-2-211

Swingley, W.D., Sadekar, S., Mastrian, S.D., Matthies, H.J., Hao, J., Ramos, H., et al. (2007) The complete genome sequence of *Roseobacter denitrificans* reveals a mixotrophic rather than photosynthetic metabolism. *J. Bacteriol.* *189*, 683-690. doi:10.1128/jb.01390-06

Wei, J., Mao, Y., Zheng, Q., Zhang, R., and Wang, Y. N. (2013) *Erythrobacter westpacificensis* sp. nov., a marine bacterium isolated from the Western Pacific. *Curr. Microbiol.* 66, 385-390.

Yurkov, V., Stackebrandt, E., Holmes, A., Fuerst, J.A., Hugenholtz, P., Golecki, J., et al. (1994) Phylogenetic positions of novel aerobic, bacteriochlorophyll *a*-containing bacteria and description of *Roseococcus* *thiosulfatophilus* gen. nov., sp. nov., *Erythromicrobium ramosum* gen. nov., sp. nov., and *Erythrobacter litoralis* sp. nov. *Int. J. Syst. Bacteriol.* 44, 427-434. doi:10.1099/00207713-44-3-427

Zheng, Q., Lin, W., Liu, Y., Chen, C., and Jiao, N. (2016) A Comparison of 14 *Erythrobacter* genomes provides insights into the genomic divergence and scattered distribution of phototrophs. *Front. Microbiol.* 7, 984. doi:10.3389/fmicb.2016.00984
